# A Single-Nucleotide Polymorphism in the Promoter of Porcine *ARHGAP24* Gene Regulates Aggressive Behavior of Weaned Pigs After Mixing by Affecting the Binding of Transcription Factor p53

**DOI:** 10.3389/fcell.2022.839583

**Published:** 2022-04-01

**Authors:** Qinglei Xu, Jing Zhao, Yanli Guo, Mingzheng Liu, Allan P. Schinckel, Bo Zhou

**Affiliations:** ^1^ College of Animal Science and Technology, Nanjing Agricultural University, Nanjing, China; ^2^ Department of Animal Sciences, Purdue University, West Lafayette, IN, United States

**Keywords:** aggressive behavior, piglet, Rho GTPase–activating protein 24, transcription factor, axon guidance, animal welfare

## Abstract

Pigs are important biomedical model animals for the study of human neurological diseases. Similar to human aggressive behavior in children and adolescents, weaned pigs also show more aggressive behavior after mixing, which has negative effects on animal welfare and growth performance. The identification of functional single-nucleotide polymorphisms (SNPs) related to the aggressive behavior of pigs would provide valuable molecular markers of the aggressive behavioral trait for genetic improvement program. The Rho GTPase–activating protein 24 (*ARHGAP24*) gene plays an important role in regulating the process of axon guidance, which may impact the aggressive behavior of pigs. By resequencing the entire coding region, partially adjacent introns and the 5′ and 3′ flanking regions, six and four SNPs were identified in the 5′ flanking region and 5′ untranslated region (UTR) of the porcine *ARHGAP24* gene, respectively. Association analyses revealed that nine SNPs were significantly associated with aggressive behavioral traits (*p* = < 1.00 × 10^–4^–4.51 × 10^–2^), and their haplotypes were significantly associated with aggressive behavior (*p* = < 1.00 × 10^–4^–2.99 × 10^–2^). The core promoter region of the *ARHGAP24* gene has been identified between −670 and −1,113 bp. Furthermore, the luciferase activity of allele A of rs335052970 was significantly less than that of allele G, suggesting that the transcriptional activity of the *ARHGAP24* gene was inhibited by allele A of rs335052970. It was identified that the transcription factor p53 bound to the transcription factor binding sites (TFBSs) containing allele A of rs335052970. In porcine primary neural cells, p53 binds to the target promoter region of the *ARHGAP24* gene, reduces its promoter transcriptional activity, and then reduces its messenger RNA (mRNA) and protein expression. The results demonstrated that the *ARHGAP24* gene had significant genetic effects on aggressive behavioral traits of pigs. Therefore, rs335052970 in the *ARHGAP24* gene can be used as a molecular marker to select the less aggressive pigs.

## Introduction

Aggressive behavior of pigs after mixing, an important animal welfare issue, causes negative impacts on the growth performance, feed conversion ratio, immunity, and meat quality, which affects the economic benefits of pig industries (Tuchscherer et al., 1998; D’Eath et al., 2010). Previous studies have shown that aggressive behavior was affected by environmental factors, such as stocking density (Arey, 1999), mixing (Greenwood et al., 2014), feeding space, and environmental enrichment (O'Connell et al., 2004). However, genetic factors also have an important impact on the aggressive behavior of pigs (Rohrer et al., 2013). Due to their complicated assessment process, aggressive behavioral traits are rarely included in traditional pig breeding selection programs. The phenotypic determination of individual animal aggressive behavior is challenging and limits the improvement of behavioral traits through genetic selection. Therefore, the identification of molecular genetic markers of aggressive behavior could contribute to the genetic selection of less aggressive pigs.

In the process of screening candidate genes for aggressive behavior in pigs, the porcine Rho GTPase–activating protein 24 (*ARHGAP24*) gene attracted our attention. The *ARHGAP24* gene encodes Rho GTPase–activating protein (RhoGAP), which stimulates the GTPase activities of the Rho family of small GTPases and terminates the binding of Rho with GTP, thus inactivating the activity of Rho family proteins (Tcherkezian and Lamarche-Vane, 2007; Muller et al., 2020). Rho GTPase family members, including RhoA, Rac, and Cdc42 proteins (Bagci et al., 2020), play important roles during the development of the nervous system (Leslie et al., 2012; Antoine-Bertrand et al., 2016). RhoA and Rac1 are regulated by GTPase-activating proteins and serve as their downstream targets (Elvers et al., 2012). Previous studies also have shown that the *ARHGAP24* gene is implicated in axon and dendrite outgrowth and branching (Nguyen et al., 2012). Meanwhile, it has been reported that aggressive behavior is associated with the axon guidance signaling pathway in humans (Viding et al., 2010; Zhang-James et al., 2019). Therefore, the *ARHGAP24* gene may play an important role in regulating the process of axon guidance, which then impacts aggressive behavior. In addition, previous studies have found that the *ARHGAP24* gene is associated with human depression (Watanabe et al., 2017) and childhood autism (Coutton et al., 2015) accompanied by aggressive behavior. The *ARHGAP24* gene is also related to the growth performance of pigs (Meng et al., 2017). However, the role of the *ARHGAP24* gene in the regulation of aggressive behavior in pigs has remained unclear.

In this study, we hypothesized that the aggressive behavior of pigs is associated with the expression and function of the *ARHGAP24* gene. We aimed to identify the functional SNPs of the *ARHGAP24* gene and investigate their molecular mechanisms for aggressive behavior regulation in weaned pigs after mixing. This research could provide valuable molecular markers of aggressive behavior for the genetic improvement of pigs.

## Materials and Methods

### Animals, Housing, and Sample Collection

This study was approved by the Animal Care and Use Committee of Nanjing Agricultural University (SYXK Su 2017-0007). A total of 500 piglets from 65 litters were selected in the Huaiyin pig breeding farm (Huaian, Jiangsu, China). The piglets were weaned at 35 days of age and moved into new empty pens with their original littermates in a nursery room 2 days before mixing. Then, nine or ten weaned pigs with the same sex and similar body weight from different litters were mixed in the pens of dimension 2.5 m × 2.2 m. The pens were equipped with slatted floors, stainless steel feeders, and nipple drinkers to allow *ad libitum* access to feed and water. The ear tissues of weaned piglets were collected, and genomic DNA was extracted by a standard phenol/chloroform method.

### Behavioral Assessment

A digital video recording system (Hikvision DS-2CE56C2P-IT3 3.6 mm; Hikvision network hard disk video recorder DS-7808HW-E1/M; Hikvision Digital Technology Co. Ltd., Hangzhou, Zhejiang, China) was used to continuously record the behavior of piglets for 72 h after mixing. A video camera was installed over each pen. To individually identify pigs in the video recording, all pigs in each pen were marked with different numbers on their back using a spray paint (7CF, Shenzhen Zhaoxin Energy Co., Ltd., Shenzhen, Guangdong, China) before mixing. The definitions of aggressive behavioral traits used were described in our previous studies (Chen et al., 2019; Tong et al., 2019) with some additional new traits. Specifically, nine indicators were used to quantify aggression, and their description and definition are shown in Table 1. A fighting behavior was recorded when it lasted for more than 3 s. For the same pair of pigs, the intervening period between each fight event was at least 8 s (Stukenborg et al., 2011).

**TABLE 1:** Description of indicators used to evaluate aggressiveness.

Trait	Description
Duration of active attack	In a fight, one pig actively bites, collides, and chases another pig which is considered to have launched an active attack (Camerlink et al., 2016). The duration is defined as the “duration of active attack”, which uses seconds as a unit of time
Frequency of active attack	As mentioned earlier, the number of active attacks is launched by pigs for 72 h after mixing, which is defined as “frequency of active attack”
Duration of being bullied	When the recipient pig suffers from biting and head-knocking performed by the aggressive pig and the recipient pig moves away without retaliation, it is regarded as being bullied (O’Malley et al., 2018). Similarly, the duration is defined as the “duration of being bullied”, which uses seconds as a unit of time
Frequency of being bullied	As mentioned earlier, the number of bullying behavior is initiated by the aggressive pig for 72 h after mixing, which is defined as the “frequency of being bullied” of the recipient pig
Duration of standoff	If two pigs stand in parallel or head-to-head, shoulder-to-shoulder, colliding, squeezing, and chasing each other during the fight and the two individuals are about equal in strength and form a single-dyadic interchange, there is no avoidance behavior (Figler et al., 2006). The duration is defined as “duration of standoff”, which uses seconds as a unit of time
Frequency of standoff	As mentioned earlier, the number of standoff behavior is launched by two pigs for 72 h after mixing, which is defined as “frequency of standoff”
CAS	The composite aggressive score (CAS) is defined as follows: CAS = frequency of active attack + 0.07 × duration of active attack (s) Shen et al. (2020)
Duration of fight	The fighting of pigs includes active attack, bullying, and standoff. The total duration of the three types of fighting behavior is defined as “duration of fight”, with seconds as the unit of time
Win	If a pig continues to attack other pigs during the fight and the attacked pig dodges, stops fighting, and tries to escape, but the aggressive pig still has intention to continue to attack, it is deemed to have won the fight (Langbein and Puppe, 2004). The number of victories achieved by the aggressive pig during fighting for 72 h after mixing is defined as “win”

### Single-Nucleotide Polymorphism Identification and Genotyping

To identify the functional SNPs of the *ARHGAP24* gene regulating the aggressive behavior of weaned pigs after mixing, specific primers (Supplementary Table S1) were used to amplify the *ARHGAP24* gene, including the coding regions, partially adjacent introns, and the 5′- and 3′-flanking regions according to the reference genome sequence of pigs (GenBank accession number: NC_010450.4). The DNA sequences contained potential SNPs of the *ARHGAP24* gene from 224 aggressive and docile pigs which were amplified by polymerase chain reactions (PCR). PCR was performed using 1.1 × T3 Super PCR Mix (TsingKe, Nanjing, Jiangsu, China), and the amplified PCR products were sequenced using the Sanger method. The DNA sequences of the porcine *ARHGAP24* gene were aligned by DNAMAN (Lynnon Biosoft, Quebec, QC, Canada) and SnapGene Viewer software (BSL Biotech LLC, Chicago, IL, United States).

### Linkage Disequilibrium Estimation and Association Analyses

The extent of LD between the identified SNPs was estimated using Haploview 4.2 (the Broad Institute of MIT and Harvard, Cambridge, MA, United States). The association analyses for aggressive behavioral traits were performed using GLIMMIX procedure of SAS 9.4 with the following generalized linear mixed model: $Y_{ijklmno}$ = µ + $G_{i}$ + $h_{j}$ + $l_{k}$ + $a_{l}$ + $b_{m}$ +c× $M_{n}$ + $e_{ijklmno}$ , where, $Y_{ijklmno}$ is the phenotypic value of the aggressive behavioral trait for each pig; μ is the overall mean; $G_{i}$ is the fixed effect of the genotype or haplotype combinations; $h_{j}$ is the fixed effect of the parity; $l_{k}$ is the fixed effect of the gender; $a_{l}$ is the individual random additive genetic effect, distributed as N (0; A ${\text{δ}}_{a}^{2}$ ), with the additive genetic variance ${\text{δ}}_{a}^{2}$ ; $b_{m}$ is the random effect of the pen; $M_{n}$ is the body weight before mixing as a covariate; c is the regression coefficient of covariate $M_{n}$ ; and $e_{ijklmno}$ is the random residual, distributed as N (0; I ${\text{δ}}_{e}^{2}$ ), with the additive genetic variance ${\text{δ}}_{e}^{2}$ .

### Promoter Prediction of the Porcine Rho GTPase–Activating Protein 24 Gene

The promoter region of the porcine *ARHGAP24* gene was predicted by Promoter 2.0 (http://www.cbs.dtu.dk/services/Promoter/) (Knudsen, 1999) and Neural Network Promoter Prediction (https://www.fruitfly.org/seq_tools/promoter.html) (Reese, 2001). Putative transcriptional binding start sites caused by the SNP mutation in the 5’ flanking or the UTR region of the *ARHGAP24* gene were predicted by using JASPAR 2020 (http://jaspar.genereg.net/) (Fornes et al., 2020) and AnimalTFDB 3.0 (http://bioinfo.life.hust.edu.cn/AnimalTFDB/#!/) (Hu et al., 2019).

### Plasmid Construction

The promoter region of the porcine *ARHGAP24* gene was amplified by PCR using Vazyme LAmp Master Mix (Vazyme Biotech, Nanjing, Jiangsu, China). Subsequently, plasmids containing variable lengths of the truncated porcine *ARHGAP24* promoter were individually amplified using different forward primers and a common reverse primer (*ARHGAP24*-P1: −33/+352; *ARHGAP24*-P2: −308/+352; *ARHGAP24*-P3: −670/+352; *ARHGAP24*-P4: −1,113/+352; *ARHGAP24*-P5: −1,572/+352; and *ARHGAP24*-P6: −1976/+352), and the primers contained MluI and XhoI recognition sequences, respectively (Supplementary Table S2). Subsequently, the amplified fragments were inserted into the multiple cloning sites of the pGL3-basic vector to generate luciferase reporter plasmids. Moreover, specific regions containing rs335052970, rs344700648, and rs339198696 were amplified using ARHGAP24-Haplotype primers contained the recognition sequences of MluI and XhoI (Supplementary Table S2). The DNA samples of piglets were amplified using primers (Supplementary Table S2) targeting the promoter region of the porcine *ARHGAP24* gene containing the p53 transcription factor binding element (PBE) motif and then cloned into the pGL3-basic vector by MluI and XhoI. The plasmid with PBE was used as a DNA template and was amplified by point mutation primer-*ARHGAP24*-PBE-MUT (Supplementary Table S2). The cDNA fragments of p53 were amplified using the primer CDS-p53 (Supplementary Table S2) and connected to the eukaryotic expression vector pcDNA3.1 (+) (pcDNA3.1-p53). The plasmid structures were sequenced to confirm the integrity of the constructed fragments.

### Cell Culture, Cell Transfection, and Luciferase Assays

Human embryonic kidney 293T (HEK 293T) cells were used for promoter activity analysis. First, HEK 293T cells were cultured in an incubator at 37°C and 5% CO_2_. The cells were plated in 12-well plates with three wells for each group. On the following day, the plasmids contained the variable length of *ARHGAP24* promoter fragments and the rs335052970 A or G allele and haplotypes were individually cotransfected into the HEK 293T cells with pRL-TK Renilla luciferase reporter vector (Promega, Madison, WI, United States) using Lipofectamine 2000 (Invitrogen, Carlsbad, CA, United States). The controls were the pGL3-basic and pGL3-control luciferase reporter gene vector. After 24 h, the cells were harvested with passive lysis buffer (Promega, Madison, WI, United States). The cell lysates were assayed for reporter gene activity using a dual-luciferase assay system (Promega, Madison, WI, United States) according to the manufacturer’s instructions.

Primary neural cells were prepared from cerebral cortices from a 1-day-old piglet, as previously described for rats (Yodoya et al., 2006). In brief, cerebral cortices were removed from the piglet’s brains. Then, the meninges and microvessels were carefully removed in ice-precooled D-Hanks’ balanced salt solution (HBSS, Gibco, Grand Island, NY, United States), and the brain tissues were minced into small pieces of about 1 mm^3^. After papain (Biofroxx, Einhausen, Germany) and DNase 1 (BioFroxx, Einhausen, Germany) were added, respectively, they were digested in an incubator at 37°C for 30 min. After the digestion was terminated with Dulbecco’s modified Eagle’s medium (DMEM, Gibco, Grand Island, NY, United States), they were sub-packed into 15-ml centrifuge tubes for centrifugation for 10 min. The porcine neural cells were cultured in DMEM supplemented with 20% fetal bovine serum (FBS, Gibco, Grand Island, NY, United States) at 37°C in a humidified atmosphere with 5% CO_2_ for 48 h. After 10 days of culture, the porcine neural cells were identified by immunofluorescence with anti–beta III tubulin (Tuj1) antibody (Abcam, Cambridge, United Kingdom). Endotoxin-free plasmids of pcDNA3.1-p53 and pcDNA3.1 were transfected into primary neural cells using Lipofectamine 3000 (Invitrogen, Carlsbad, CA, United States). The siRNA of p53 and ARHGAP24 were designed and chemically synthesized by Shanghai Jima Pharmaceutical Technology Co., Ltd. The primer sequence is shown in Supplementary Table S2. Either scrambled siRNA or p53 siRNA plasmids was transfected into primary neural cells using Lipofectamine 3000 (Invitrogen, Carlsbad, CA, United States) according to the manufacturer’s instructions.

### RNA Isolation and Quantitative Reverse Transcription-PCR

The cells were harvested at day 1 post transfection. The total RNA of porcine neural cells was extracted by using TRIzol reagent (Invitrogen, Carlsbad, CA, United States) according to the manufacturer’s protocol. The purity of RNA was detected by the NanoDrop 2000 (Thermo Fisher Scientific, Fremont, CA, United States). To quantify the mRNA expression level of *ARHGAP24* and *p53*, total RNA was reverse-transcribed onto cDNA using the HiScript III RT SuperMix (Vazyme Biotech, Nanjing, Jiangsu, China). The RT-qPCR was performed on the Quantum Studio 5 quantitative PCR instrument (Applied Biosystems, Foster, CA, United States) using SYBR Green Master Mix (Vazyme Biotech, Nanjing, Jiangsu, China) and specific primers (Supplementary Table S2). Relative expression levels were calculated by using the 2^−ΔΔCt^ method (Livak and Schmittgen, 2001). The coding gene expression levels were normalized to the expression of *GAPDH*. For the RT-qPCR reaction, each treatment had at least three biological replicates.

### Western Blotting

The cell protein lysates were harvested using 200 µL ice-cold radioimmunoprecipitation assay (RIPA) lysis buffer (Beyotime, Shanghai, China) with 1% phenylmethyl sulfonyl fluoride (PMSF, Beyotime, Shanghai, China). Total protein extracts were separated on 4–20% SDS–PAGE gels (GenScript Biotech, Nanjing, Jiangsu, China) and then blotted onto a polyvinylidene fluoride (PVDF) membrane (Bio-Rad, Hercules, CA, United States). After blocking with QuickBlock™ blocking buffer (Beyotime, Shanghai, China) for 30 min, the PVDF membranes were incubated overnight with the following primary antibodies: immunoreactive proteins were detected with a rabbit polyclonal antibody for anti-P53 (1:1,000; AF0879, Affinity, China), anti-ARHGAP24 (1:1,000; DF9858, Affinity, China), and anti-GAPDH (1:5,000; AF7021, Affinity, China). The appropriate antirabbit secondary antibody (1:8,000; S0001, Affinity, China) was used to incubate the membranes. The ECL peroxidase color development kit (Vazyme, Nanjing, Jiangsu, China) was used in chromogenic reaction according to the manufacturer’s instructions. The protein band visualization was performed by the ChemiDoc XRS + System (Bio-Rad, Hercules, CA, United States). The band density was analyzed using ImageJ software.

### Statistical Analyses

Data were analyzed using SAS 9.4 (SAS Institute Inc., Cary, NC, United States). Chi-square tests were applied to analyze the difference in allele frequency between the most aggressive and least aggressive pigs. The behavioral data were analyzed using the GLIMMIX procedure with a model option DIST = EXPO. The relative fluorescence activity value was normalized by the negative control pGL3-basic. The significance of luciferase activity statistics was analyzed by unpaired two-sided student’s *t*-test and one-way ANOVA. The results were presented as mean ± standard error of the mean (SEM), and *p* < 0.05 was considered statistically significant.

## Results

### Identification of Single-Nucleotide Polymorphisms in Porcine Rho GTPase–Activating Protein 24 Gene

A total of 10 SNPs were identified by sequencing on the entire coding region, 5′- and 3′-flanking regions of the porcine *ARHGAP24* gene in 178 pigs (Table 2). In total, six SNPs (rs339198696, rs344700648, rs335052970, rs344498203, rs323776551, and rs342083908) were located in the 5′-flanking region, four SNPs (rs333053350, rs342210686, rs328435752, and rs787973778) were located in the 5′-UTR of the porcine *ARHGAP24* gene, and five SNPs (rs344700648, rs335052970, rs323776551, rs342083908, and rs787973778) showed significant difference (*p* < 0.05) in allele frequencies between the most aggressive and least aggressive pigs.

**TABLE 2:** Allele frequencies of single-nucleotide polymorphisms (SNPs) in the porcine *ARHGAP24* gene.

SNPs	Location	Mutation type	Allele	Aggressive/docile frequency	χ^2^	*p-*value
rs339198696	5′-flanking region	A > C	A	0.54/0.71	1.39	0.238
			C	0.46/0.29		
rs344700648	5′-flanking region	T > A	T	0.00/0.25	6.86	0.009**
			A	1.00/0.75		
rs335052970	5′-flanking region	G > A	G	0.92/0.50	10.08	0.001**
			A	0.08/0.50		
rs344498203	5′-flanking region	C > G	C	0.88/0.71	2.02	0.155
			G	0.13/0.29		
rs323776551	5′-flanking region	C > A	C	0.21/0.50	4.46	0.035*
			A	0.79/0.50		
rs342083908	5′-flanking region	G > A	G	0.88/0.58	5.17	0.023*
			A	0.13/0.42		
rs333053350	5′ UTR	G > T	G	0.79/0.54	3.38	0.066
			T	0.21/0.46		
rs342210686	5′ UTR	G > A	G	0.58/0.33	3.02	0.082
			A	0.42/0.67		
rs328435752	5′ UTR	A > G	A	0.79/0.54	3.38	0.066
			G	0.21/0.46		
rs787973778	5′ UTR	A > C	A	0.83/0.54	4.75	0.029*
			C	0.17/0.46		

Note: χ^2^: chi-square value; * statistically significant, *p < 0.05 and **p < 0.01.

### Association Analyses Between the Genotype of the Rho GTPase–Activating Protein 24 Gene and Aggressive Behavior in Pigs

**FIGURE 1:**
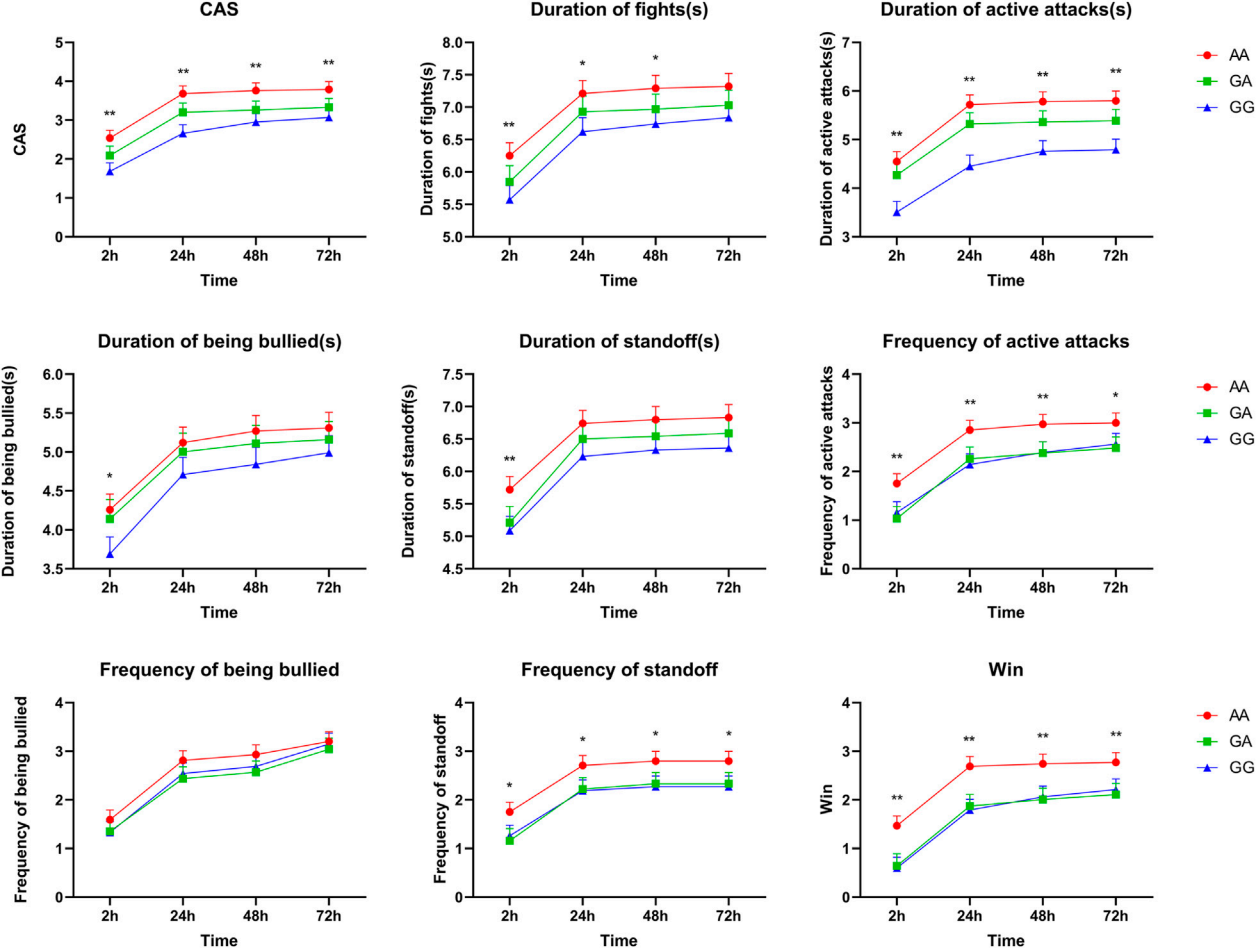
Associations of SNP rs335052970 in the *ARHGAP24* gene with aggressive behavioral traits at the first 2, 24, 48, or 72 h after mixing in weaned pigs (LSM ± SE). *p < 0.05 and **p < 0.01 indicate that the difference is significant.

Association analyses between genotype and aggressive behaviors during the first 2, 24, 48, or 72 h after mixing are presented in Supplementary Table S3. A total of three SNPs (rs339198696, rs344700648, and rs335052970) in 5′-flanking regions were significantly associated with aggressive behaviors during the first 2, 24, 48, or 72 h after mixing (*p* < 0.05). Interestingly, all SNPs had a strong association with multiple aggressive behaviors during the first 2 h after mixing. Moreover, CAS, duration of active attacks, frequency of active attack, frequency of standoff, and win were greater in the pigs with the mutant AA genotype of rs335052970 than those in the pigs with the wild GG genotype during the first 2, 24, 48, or 72 h after mixing (*p* < 0.05, Figure 1). Similarly, four SNPs (rs333053350, rs342210686, rs328435752, and rs787973778) in the 5′-UTR were significantly associated with the duration of active attacks and duration of standoff (*p* < 0.05) (Supplementary Table S3 for details).

### Association Analyses Between the Haplotype of Rho GTPase–Activating Protein 24 Gene and Aggressive Behavior in Pigs

**FIGURE 2:**
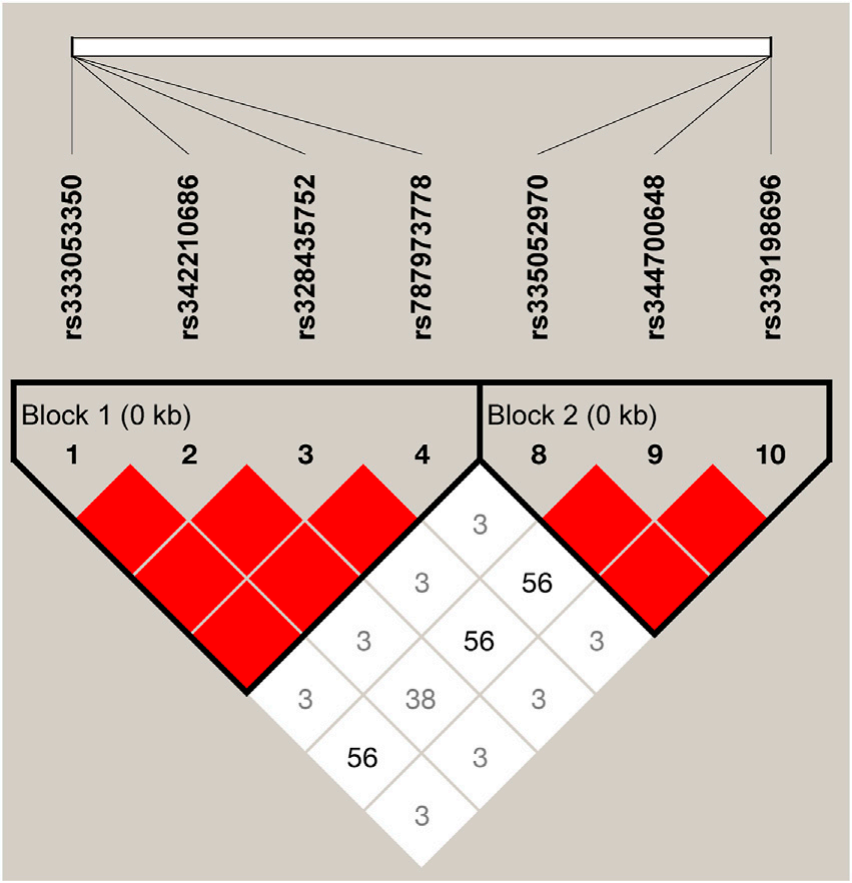
LD among the SNPs in the porcine *ARHGAP24* gene (D’ = 0.03–1.00). It is to be noted that the blocks indicate haplotype blocks and the text before the horizontal numbers is the SNP name. The values in boxes are pairwise SNP correlations (D’), while bright red boxes without numbers represent complete LD (D’ = 1).

We estimated the LD among the 10 SNPs of the *ARHGAP24* gene using Haploview 4.2. A total of seven SNPs (rs333053350, rs342210686, rs328435752, rs787973778, rs335052970, rs344700648, and rs339198696) were highly linked (D′ > 0.69; Figure 2) in two haplotype blocks. The first haplotype block (block 1) consisted of three haplotypes: H1 (GGAA), H2 (TAGC), and H3 (GAAA). The second haplotype block (block 2) consisted of three haplotypes: H1 (AAC), H2 (GAA), and H3 (GTA). The two haplotype blocks were significantly associated with aggressive behavior (*p* < 0.05) (Supplementary Table S4). In the haplotype block 1, pigs with haplotype H1 (GGAA) were more aggressive than pigs with haplotype H2 (TAGC) or H3 (GAAA). Similarly, pigs with haplotype H1 (AAC) in haplotype block 2 were more aggressive than those with haplotype H2 (GAA) or H3 (GTA) (*p* < 0.05).

### Promoter Prediction and Identification of the Porcine Rho GTPase–Activating Protein 24 Gene

**FIGURE 3:**
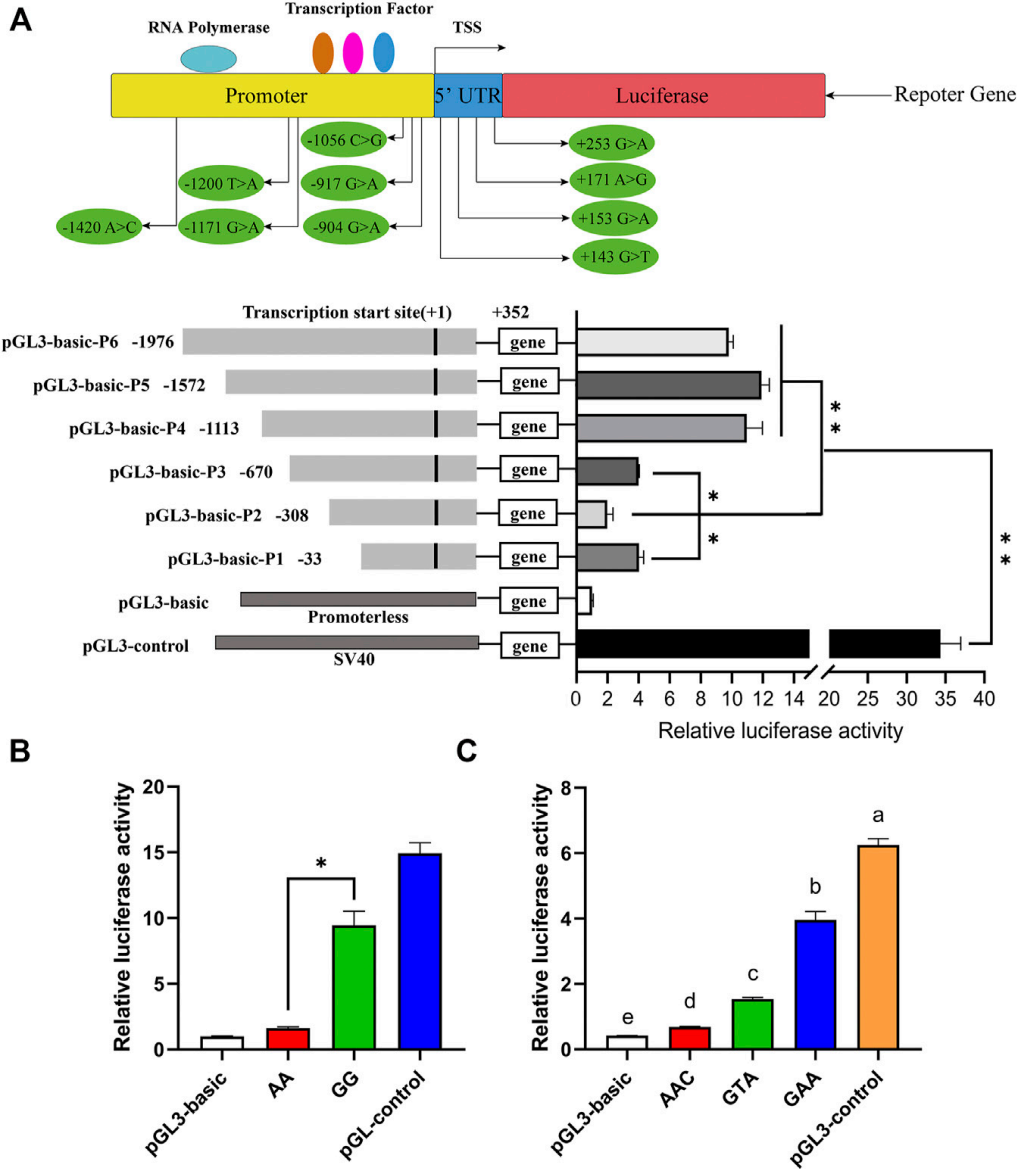
Luciferase assays for porcine *ARHGAP24* promoter activity analyses. PGL3-basic as a negative control and PGL3-control as a positive control. **(A)** Gene promoter diagram and the location of SNPs in the promoter region and the 5′ UTR. A total of six luciferase reporter plasmids expressing successive truncations of the *ARHGAP24* promoter sequence were constructed and transfected into HEK 293T cells. **(B)** Luciferase reporter gene assays of porcine *ARHGAP24* alleles contained rs335052970 (−744G > A). **(C)** Luciferase activities of plasmids contained three haplotypes of the porcine *ARHGAP24* gene. The relative luciferase activity values represent the mean ± SEM of three independent experiments. Statistical differences in luciferase activity were assessed using the one-way ANOVA; *p < 0.05 and **p < 0.01. Different letters (a, b, c, *etc*.) indicate that the difference is significant (*p* < 0.05).

A total of two promoter regions (−1,364/−1,314 bp and +89/+139 bp) and three transcription initiation sites (−1,700, −800, and +100 bp) of the porcine *ARHGAP24* gene were predicted by Promoter 2.0 and Neural Network Promoter Prediction. The transcription factor potential binding sites for RUNX2, RREB1, IRF2, IRF1, p53, CREBBP, POLR3A, and GLI1 were predicted in the 5′ flanking region of the porcine *ARHGAP24* gene (Supplementary Table S5). In the promoter activity analyses, the luciferase activity of plasmids that contained the promoter fragments of the *ARHGAP24* gene was greater than that of the pGL3-basic plasmid (*p* < 0.01) but less than that of the pGL3-control plasmid (*p* < 0.01). Moreover, the luciferase activity of pGL3-basic-P4, pGL3-basic-P5, and pGL3-basic-P6 was greater than that of pGL3-basic-P1, pGL3-basic-P2, and pGL3-basic-P3 (*p* < 0.01). The luciferase activity of pGL3-basic-P3 and pGL3-basic-P1 was greater than that of pGL3-basic-P2 (*p* < 0.05) (Figure 3A). These results revealed that the core promoter region of the *ARHGAP24* gene is located between −670 and −1,113 bp, whereas a negative regulatory promoter region is located between −308 and −33 bp.

### Promoter Activity Analyses of the Porcine Rho GTPase–Activating Protein 24 Gene

The luciferase activity was greater in plasmids that contained the G allele of rs335052970 than that of plasmids containing the A allele (*p* < 0.01) (Figure 3B). Moreover, there are three linked SNPs (rs335052970, rs344700648, and rs339198696) in the core promoter region (−670/−1,113 bp) of the *ARHGAP24* gene. They form only three haplotypes: H1 (AAC), H2 (GAA), and H3 (GTA). The luciferase activity of plasmids that contained the haplotypes of the core promoter region was greater than that of pGL3-basic but less than that of the pGL3-control (*p* < 0.0001). The relative luciferase activity of plasmids that contained haplotype GAA was the greatest, while that of plasmids that contained haplotype AAC was the least (*p* < 0.01) (Figure 3C). It indicates that the site affecting promoter activity is rs335052970 (−744G > A).

### Transcription Factor p53 Regulates Rho GTPase–Activating Protein 24 Gene Expression in Porcine Neural Cells

**FIGURE 4:**
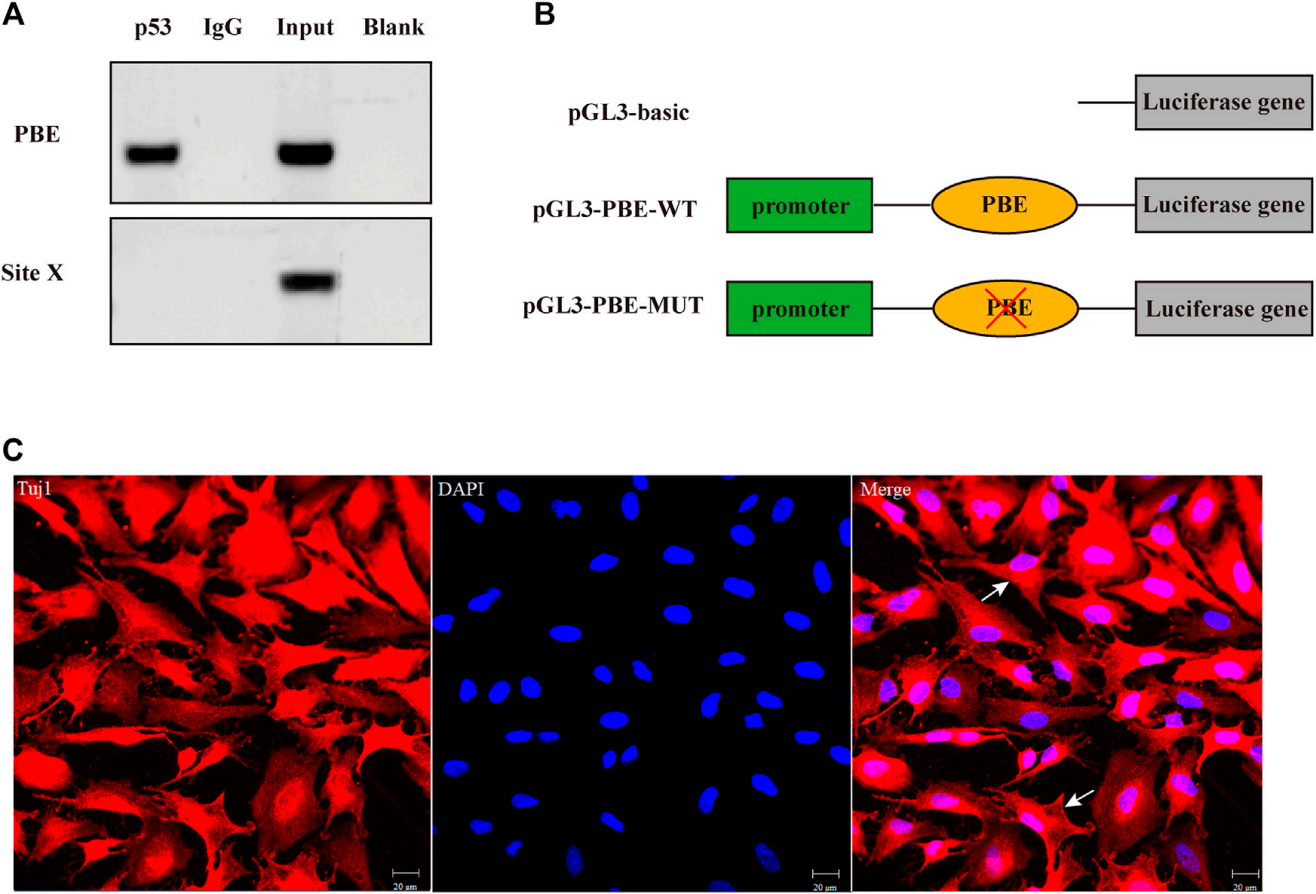
Transcription factor p53 directly targeted the binding element (PBE) motif of the *ARHGAP24* gene in porcine neural cells. **(A)** Confirmation of the direct interaction between the p53 and *ARHGAP24* promoter. ChIP-qPCR assay was performed with IgG as the negative control. Site X, a negative control locus, input, and total DNA from untreated cells. **(B)** Construction of luciferase activity reporter vectors containing wild-type (WT) and mutant-type (MUT) PBE on the promoter of the *ARHGAP24* gene. Blue boxes represent the luciferase gene; green boxes represent pGL-3 promoter; orange ovals represent PBE; and red fork represents mutation. **(C)** Immunofluorescence identification of porcine primary neural cells. Immunofluorescence staining of porcine neural cells with Tuj1 (red) and DAPI (blue); the white arrow shows the primary porcine nerve cells; Scale bars represent 20 μm.

**FIGURE 5:**
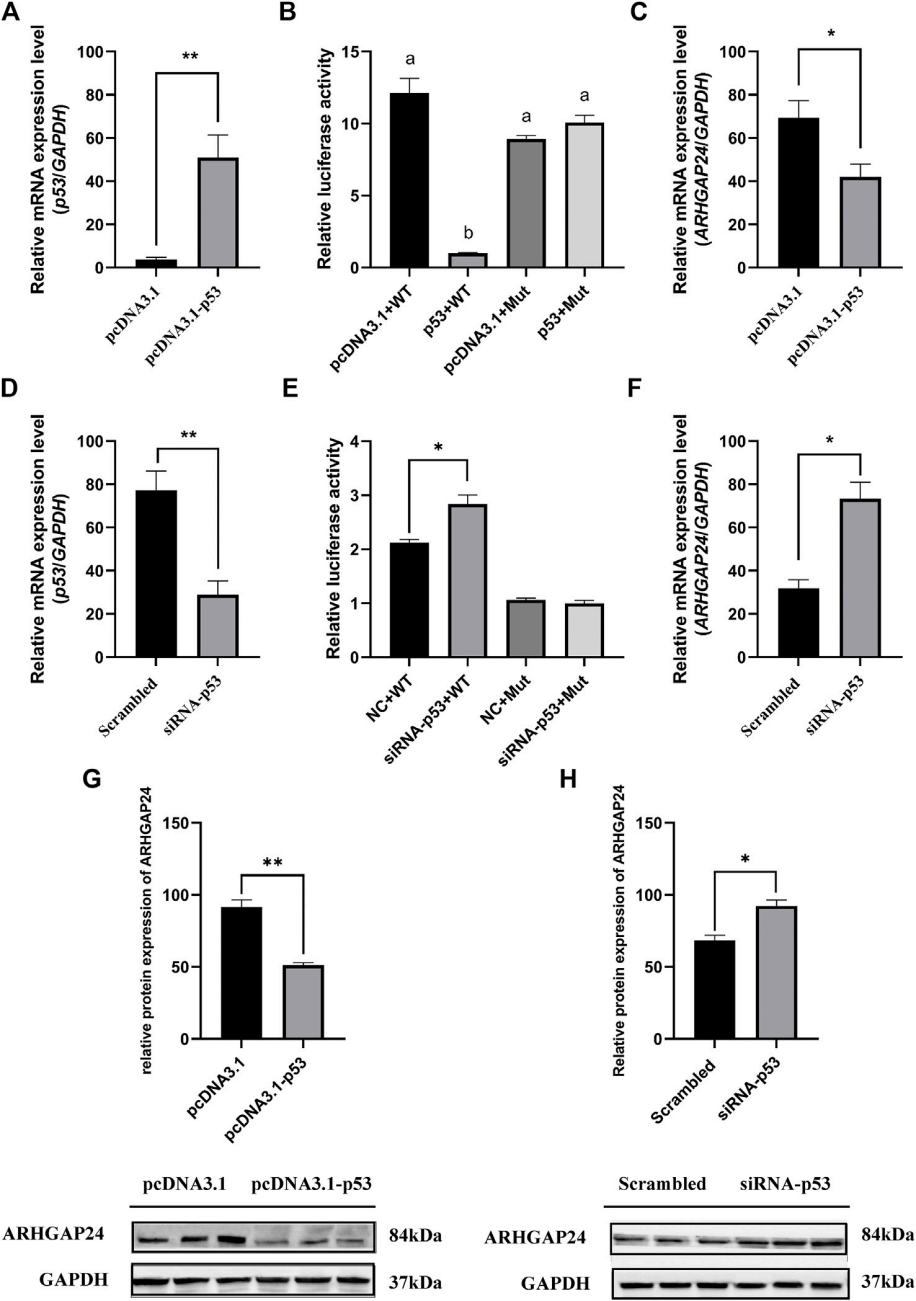
Transcription factor p53 regulates *ARHGAP24* gene expression in porcine neural cells. **(A)** mRNA level of p53 after p53 overexpression. **(B)** mRNA level of p53 after p53 knockdown. **(C)** Luciferase activity of the *ARHGAP24* promoter region after p53 overexpression **(D)** Luciferase activity of the *ARHGAP24* promoter region after p53 knockdown **(E)** mRNA level of *ARHGAP24* after p53 overexpression. **(F)** mRNA level of *ARHGAP24* after p53 knockdown. **(G)** Western blot analyses of ARHGAP24 protein expression in porcine neural cells transfected with pcDNA3.1-p53 and pcDNA3.1 (+). **(H)** Western blot analyses of ARHGAP24 protein expression in porcine neural cells transfected with the scrambled and siRNA-p53 groups. The protein levels were normalized to GAPDH. *p < 0.05 and **p < 0.01. Different letters (a, b, c, *etc*.) before the columns indicate that the difference is significant (*p* < 0.05)

The effects of SNP rs335052970 (−744G > A) on the transcription factor binding sites (TFBSs) were predicted using the Animal TFDB online website. The allele A of rs335052970 was found to be located in the potential binding sequence of the transcription factor p53 (TP53) (Supplementary Table S5). To verify the binding sequence of the transcription factor p53 in the upstream region of the *ARHGAP24* gene containing SNP rs335052970, a chromatin immunoprecipitation (ChIP) assay was used to demonstrate that p53 binds to the transcription factor binding element (PBE) motif directly *in vivo* (Figure 4A). To investigate whether p53 regulates the expression of *ARHGAP24* through the PBE site, we cloned the PBE site into a pGL3 vector (Promega, United States) to construct PBE-allele A (pGL3-WT) and PBE-allele G (pGL3-MUT) reporter vectors (Figure 4B). Reporter vectors and p53 overexpression vector (pcDNA3.1-p53) were cotransfected into porcine neural cells. The results of immunofluorescence identification are shown in Figure 4C. Porcine primary neural cells treated with TuJ1 antibody showed red fluorescence, indicating that TuJ1 detection is positive, the cell neurites are connected with each other, and the dendritic contour is clearly visible. The mRNA expression level of *p53* in the pcDNA3.1-p53 group was greater than that in the control group (*p* < 0.05) (Figure 5A). The mRNA expression level of *p53* in the siRNA-p53 group was less than that in the scrambled group (*p* < 0.05) (Figure 5B). The luciferase activity of the pcDNA3.1-WT group was less than that of the pcDNA3.1-MUT group (*p* < 0.05) when p53 was overexpressed (Figure 5C). Moreover, the luciferase activity of the siRNA-p53 group was greater than that of the control group (*p* < 0.05) (Figure 5D). The overexpression of p53 reduced the mRNA and protein expression level of *ARHGAP24* (*p* < 0.01) (Figures 5E,G), but interfering p53 increased the mRNA expression level of *ARHGAP24* (*p* < 0.01) (Figures 5F,H).

### Transcription Factor p53 Regulates Aggression in Pigs Through the Axon Guidance Pathway

**FIGURE 6:**
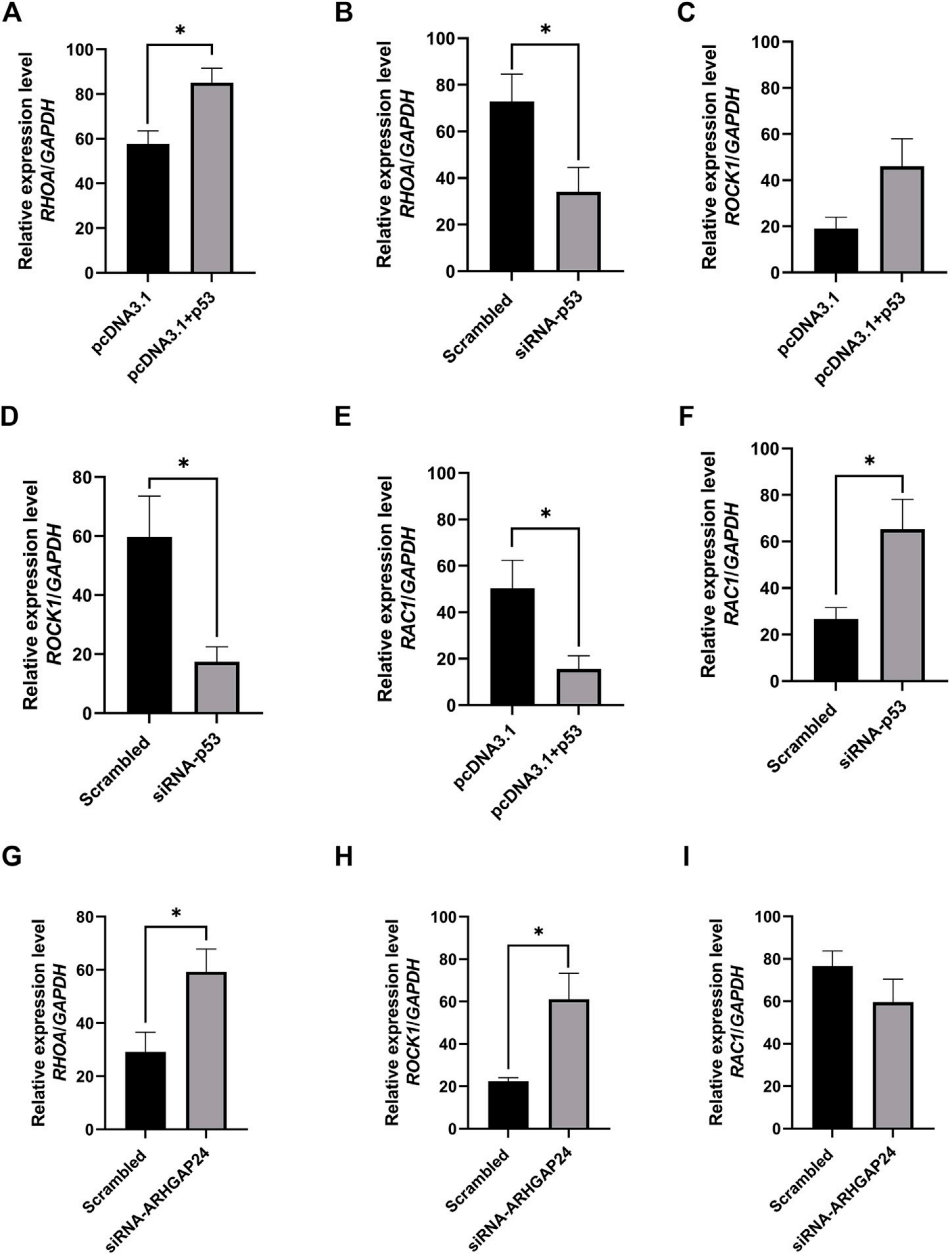
Transcription factor p53 regulates axon guidance pathway–related gene expression in porcine neural cells. **(A)** mRNA level of *RHOA* after p53 overexpression. **(B)** mRNA level of *RHOA* after p53 knockdown. **(C)** mRNA level of *ROCK1* after p53 overexpression **(D)** mRNA level of *ROCK1* after p53 knockdown **(E)** mRNA level of *RAC1* after p53 overexpression. **(F)** mRNA level of *RAC1* after p53 knockdown. **(G)** mRNA level of *RHOA* after *ARHGAP24* knockdown. **(H)** mRNA level of *ROCK1* after *ARHGAP24* knockdown. **(I)** mRNA level of *RAC1* after *ARHGAP24* knockdown. *p < 0.05 and **p < 0.01. Scrambled: a negative control for siRNA as a scrambled sequence of the siRNA target sequence.

In order to explore how the signal pathway involved in the *ARHGAP24* gene regulates aggressive behavior and the expression of related genes in the signal pathway when p53 is overexpressed or inhibited, we connected the eukaryotic expression vector pcDNA3.1 (+) (pcDNA3.1-p53) and chemically synthesized siRNA-p53 and siRNA-ARHGAP24 and then transfected into porcine neural cells to detect the expression level of related genes in the axon guidance pathway. The mRNA expression level of *RHOA* in the pcDNA3.1-p53 group was greater than that in the control group (*p* < 0.05) (Figure 6A), while the mRNA expression level of *RHOA* in the siRNA-p53 group was less than that in the scrambled group (*p* < 0.05) (Figure 6B). Similarly, the mRNA expression level of *ROCK1* in the pcDNA3.1-p53 group had an increased tendency than that in the control group (*p* = 0.0567) (Figure 6C), while the mRNA expression level of *ROCK1* in the siRNA-p53 group was less than that in the scrambled group (*p* < 0.05) (Figure 6D). By contrast, the mRNA expression level of *RAC1* in the pcDNA3.1-p53 group was less than that in the control group (*p* < 0.05) (Figure 6E), while the mRNA expression level of *RAC1* in the siRNA-p53 group was greater than that in the scrambled group (Figure 6F). Moreover, the mRNA expression level of *RHOA* and *ROCK1* in the siRNA-ARHGAP24 group was greater than that in the scrambled group (*p* < 0.05) (Figures 6G,H). However, the mRNA expression level of *RAC1* was not different between the siRNA-ARHGAP24 and scrambled groups (Figure 3I).

## Discussion

To our knowledge, the *ARHGAP24* gene encodes a GTPase-activating protein. RhoGAPs are the important negative regulators of the Rho signaling pathway (Wang et al., 2019). Studies have also revealed that several members of the Rho family of GTPase activators have neuronal functions, including regulating dendritic morphology and synaptic plasticity (Ramakers, 2002). *ARHGAP24* has been found to be a genetic marker to distinguish patients with major depression from healthy people (Watanabe et al., 2017). In this study, we first demonstrated that SNPs in the 5′-flanking region of the *ARHGAP24* gene were associated with several aggressive behavioral traits. Among them, the difference of aggression among individuals with different genotypes at SNP rs335052970 is the most significant, which suggests that this SNP is worthy of further investigation. Four SNPs (rs333053350, rs342210686, rs328435752, and rs787973778) in the 5′-UTR were also significantly associated with aggressive behavioral traits. It is interesting that pigs with wild genotypes of this four linked SNPs in the *ARHGAP24* gene were more aggressive than pigs with mutant genotypes. Next, we predicted the changes of TFBSs caused by them. The TFBSs for MECP2, REST, and NOTCH1 contain one of the four SNPs in the 5′-UTR. Previous studies showed that the genes activated by MECP2 caused many neuropsychiatric diseases (Chahrour et al., 2008). As a neural specific target, the transcription factor REST precisely regulated the transcription in the process of neuronal differentiation and development (Monestime et al., 2019). We herein hypothesized that MECP2, REST, and NOTCH1 could upregulate/downregulate the expression of *ARHGAP24* to affect the aggressive behavioral traits. In addition, haplotypes were used as markers in association analysis to explain important genetic variation (Nothnagel and Rohde, 2005). LD was used to locate causal mutation sites that could not be precisely located by simple single-marker association (Hagenblad et al., 2004). Association analyses between haplotypes and aggressive behavior traits revealed that two haplotype blocks were all significantly associated with aggressive behavioral traits, which is consistent with the association between the SNPs and aggressive behavior trait in weaned pigs after mixing.

A promoter is necessary for the initiation of gene transcription and one of the upstream *cis*-acting elements for gene expression regulation (Civas et al., 2006; Yaniv, 2014). A core promoter initiates transcription, including transcription initiation sites (TSS) and upstream elements (Smale and Kadonaga, 2003). Based on luciferase activity analyses, the porcine *ARHGAP24* gene had not only a positive regulatory promoter region (from −670 to −1,113 bp) but also a negative regulatory promoter region located (from −308 to −33 bp). The core promoter region has specific transcription factor binding sites and initiates the expression of downstream genes (Lubliner et al., 2015). In addition, the SNPs located in the core promoter region affected mRNA transcription by affecting the binding to transcription factors (Zubenko and Hughes, 2009). A previous study has shown that SNPs in the 5′-UTR of *FGF13* interfered with the translation process of *FGF13* and led to defects in the brain development and cognitive functions (Pan et al., 2021). In the present study, three SNPs (rs335052970, rs344700648, and rs339198696) in the core promoter region were in LD. Moreover, the relative luciferase activity of plasmids with haplotype AAC was the least than those with the other two haplotypes. A possible reason for lower transcriptional activity is that the allele A of rs335052970 present in the haplotype AAC promoter sequence promotes the potential binding site of the transcriptional repressors. Meanwhile, the luciferase activity was greater in plasmids with genotype GG than that of plasmids with genotype AA of rs335052970, implying that the promoter with allele G of rs335052970 might have higher transcriptional activity than the promoter with allele A. In conclusion, in view of the significant genetic effect on the aggressive behavior of SNP rs335052970 located in the core promoter region, it may regulate the expression of the *ARHGAP24* gene by affecting promoter activity. Further research is needed to analyze how SNPs rs335052970 regulate aggressive behavior in pigs.

Previous studies revealed that SNPs located in the core promoter region changed the transcription factor binding sites (An et al., 2011; Vega et al., 2018). Transcription factors can activate or inhibit gene expression, which could result in a change of phenotype (Ameur et al., 2009; Kim et al., 2015). A previous study has shown that transcription factor YY1 binds to the promoter region of the *Stx1a* gene related to synaptic transmission and neurodevelopmental disorders and negatively regulates its transcription in a cell/tissue-specific manner (Nakayama et al., 2021). In the present study, the allele A of rs335052970 located in the core promoter region of the *ARHGAP24* gene was predicted to invent the TFBSs for p53. Subsequently, a ChIP analysis demonstrated that p53 directly binds to the transcription factor binding element (PBE) motif containing allele A of rs335052970 *in vitro*. p53, a tumor suppressor gene (Boutelle and Attardi, 2021), is well-known for its functions as a transcription factor, which mediates transcriptional activation (Olivero et al., 2020) or repression (Wang et al., 2010). In the present study, the mRNA and protein expression level of *ARHGAP24* was decreased after the overexpression of p53. Furthermore, the mRNA and protein expression level of *ARHGAP24* was increased by interfering p53. A previous study presented that p53 acted as a repressor to downregulate PRR11-SKA2 to inhibit tumor formation (Wang et al., 2017). Furthermore, p53 acts as a transcription factor, represses the transcription of the *PINK1* gene and then inhibits autophagy (Goiran et al., 2018), and represses antiapoptotic target genes (Castrogiovanni et al., 2018), which is similar to our present study. In addition, a recent study found that p53 may be a central regulator of neurodegeneration (Maor-Nof et al., 2021). Therefore, we speculated that p53 binds to TFBSs containing allele A of rs335052970 in the core promoter region of the *ARHGAP24* gene, reduces the transcriptional activity of the promoter, and then inhibits the mRNA and protein expression level of the *ARHGAP24* gene in porcine neural cells.

**FIGURE 7:**
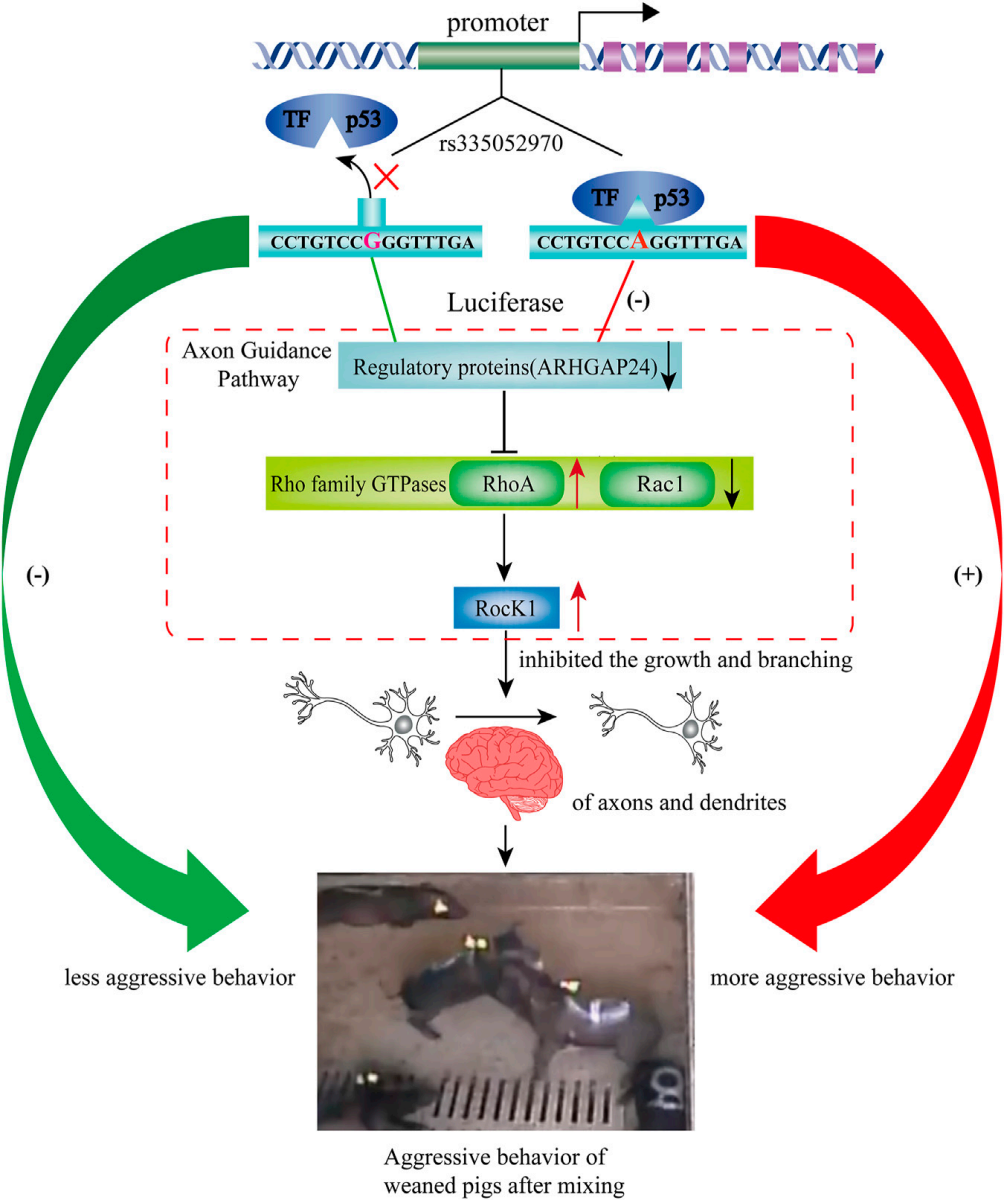
Based on the results, we hypothesized a regulating pathway by rs335052970 on the aggressive behavior of weaned pigs after mixing. Based on the results, we hypothesized a regulating pathway by rs335052970 in the *ARHGAP24* gene on the aggressive behavior of pigs. In this diagram, rs335052970 binds to the transcription factor p53 to form a complex that regulates *ARHGAP24* gene expression. Specifically, the promoter with allele A is more capable of binding to the transcriptional repressor p53 and downregulates *ARHGAP24* gene expression. Since RhoA and RocK1 are the downstream targets of ARHGAP24, p53 also activates the expression of RhoA and Rock1. This might reduce the growth and branching of axons and dendrites, which make pigs more aggressive after weaning. Consequently, the promoter with allele A of rs335052970 upregulates the aggression of weaned pigs after mixing.

The pathways regulating aggressive behavior include the G-protein–coupled receptor (GPCR) signaling pathway, axon guidance, and ERK/MAPK signaling (Zhang-James et al., 2019). The small GTPase Rho, including RhoA, Rac, and Cdc42, as downstream regulators of RhoGAPs regulates the development of the nervous system by participating in the axon guidance pathway (Antoine-Bertrand et al., 2016). Rho kinase (ROCK), a downstream target of small GTPase Rho, is associated with a variety of neural functions, such as dendritic development and axon extension (Fujita and Yamashita, 2014). In the present study, the mRNA expression level of *RHOA* was increased after the overexpression of p53, while the mRNA expression level of *RHOA* and *ROCK1* was decreased by interfering p53. While, the mRNA expression level of *RAC1* was decreased after the overexpression of p53, it was increased by interfering p53. In general, Rac1 and Cdc42 are the positive regulators of axon growth and guidance, while RhoA is a negative regulator (Gonzalez-Billault et al., 2012). Moreover, the mRNA expression level of *RHOA* and *ROCK1* was greater than that in the scrambled group when *ARHGAP24* was inhibited in the present study. It has been reported that p53 was transcriptionally activated and participated in neural growth factor–mediated neurite growth (Brynczka et al., 2007; Brynczka and Merrick, 2008). Meanwhile, the deregulation of the *ARHGAP24* gene inhibited the growth and branching of axons and dendrites (Nguyen et al., 2012). Repeated stress in rats resulted in atrophy of dendrites in hippocampal and medial prefrontal cortex neurons and increased aggression (Miller and McEwen, 2006). Therefore, p53 might reduce the axonal outgrowth and dendritic arborization by inhibiting the expression of the *ARHGAP24* gene, which makes pigs more aggressive after weaning (Figure 7). Thus, SNP rs335052970 may be a potential causal mutation agent of porcine aggressive behavioral traits. It changes the transcriptional activity of the *ARHGAP24* gene and regulates gene expression of axon guidance in combination with the transcription factor p53. However, further functional studies are needed to verify how the transcription factors affect the aggressive behavior of pigs.

In conclusion, our results revealed the significant genetic effects of the *ARHGAP24* gene on aggressive behavioral traits in weaned pigs after mixing. The functional SNP can be used for the genetic selection for less aggressive pigs. In addition, rs335052970 was highlighted as a functional mutation for aggressive behavioral traits that changed the transcriptional activity of the *ARHGAP24* gene by affecting the binding of the transcription factor p53. Furthermore, functional verification is needed to find further scientific evidence on the regulation mechanism of the *ARHGAP24* gene on aggressive behavioral traits in pigs.

## Data Availability

The authors acknowledge that the data presented in this study must be deposited and made publicly available in an acceptable repository, prior to publication. Frontiers cannot accept a article that does not adhere to our open-data policies.

## Abbreviations

ARHGAP24, Rho GTPase-activating protein 24; ChIP, chromatin immunoprecipitation; GPCR, G-protein-coupled receptor; HEK 293T, human embryonic kidney 293T; PBE, p53 transcription factor binding element; PCR, polymerase chain reactions; RhoGAP, GTPases activating protein; SNPs, single-nucleotide polymorphisms; TP53, transcription factor p53; TSS, transcription initiation sites; ROCK, Rho kinase; and UTR, untranslated region.
