# Supplementary material for: A Single-Nucleotide Polymorphism in the Promoter of Porcine ARHGAP24 Gene Regulates Aggressive Behavior of Weaned Pigs After Mixing by Affecting the Binding of Transcription Factor p53
Source: Front Cell Dev Biol. 2022 Apr 1;10:839583. doi: 10.3389/fcell.2022.839583 (PMC9010951; doi:10.3389/fcell.2022.839583)
Supplement: Supplementary file 1 [file Table4.DOC]

# Supplementary Tables

**Table S4.** Associations of the SNPs in the *ARHGAP24* gene with aggressive behavioral traits at 2 h, 24h, 48h, 72h after mixing in pigs (LSM ± SE).

| SNP | Genotype (No.) | CAS | Duration of fights(s) | Duration of active attacks(s) | Duration of being bullied(s) | Duration of standoff(s) | Frequency of active attacks | Frequency of being bullied | Frequency of standoff | Win |
| --- | --- | --- | --- | --- | --- | --- | --- | --- | --- | --- |
| 2 h after mixing | | | | | | | | | | |
| rs339198696 | AA (52) | 1.74±0.23^b^ | 5.43±0.23^b^ | 3.75±0.24^b^ | 3.19±0.23^b^ | 4.97±0.24^b^ | 1.01±0.23^b^ | 1.03±0.24^b^ | 1.05±0.23^b^ | 0.58±0.23^b^ |
|  | CA (24) | 1.71±0.27^b^ | 5.89±0.27^ab^ | 3.72±0.27^b^ | 3.98±0.27^a^ | 5.52±0.28^a^ | 1.00±0.27^b^ | 1.20±0.27^b^ | 1.40±0.27^ab^ | 0.48±0.27^b^ |
|  | CC (77) | 2.46±0.19^a^ | 6.14±0.19^a^ | 4.49±0.19^a^ | 4.28±0.19^a^ | 5.58±0.19^a^ | 1.66±0.19^a^ | 1.64±0.19^a^ | 1.66±0.19^a^ | 1.33±0.19^a^ |
|  | *p* Value | 0.0004** | 0.0018** | 0.0004** | <.0001** | 0.0062** | 0.0017** | 0.0051** | 0.0083** | <.0001** |
| rs344700648 | AA (78) | 2.27±0.19 | 6.02±0.19^ab^ | 4.17±0.19^a^ | 4.04±0.19 | 5.52±0.19^a^ | 1.63±0.19^a^ | 1.63±0.19^a^ | 1.65±0.19^a^ | 1.27±0.19^a^ |
|  | AT (32) | 2.31±0.25 | 6.24±0.25^a^ | 4.52±0.25^a^ | 3.95±0.25 | 5.79±0.25^a^ | 1.25±0.25^ab^ | 0.87±0.26^b^ | 1.27±0.25^ab^ | 1.02±0.25^ab^ |
|  | TT (40) | 1.95±0.24 | 5.69±0.24^b^ | 4.01±0.24^b^ | 4.14±0.24 | 5.05±0.24^b^ | 1.10±0.24^b^ | 1.20±0.24^ab^ | 1.18±0.24^b^ | 0.62±0.23^b^ |
|  | *p* Value | 0.2562 | 0.0791 | 0.1175 | 0.7844 | 0.0098** | 0.0391* | 0.0027** | 0.0624 | 0.0103* |
| rs335052970 | AA (76) | 2.54±0.20^a^ | 6.25±0.20^a^ | 4.55±0.20^a^ | 4.26±0.20^a^ | 5.72±0.20^a^ | 1.75±0.20^a^ | 1.59±0.20 | 1.75±0.20^a^ | 1.47±0.20^a^ |
|  | GA (38) | 2.09±0.24^b^ | 5.85±0.25^ab^ | 4.27±0.25^a^ | 4.14±0.25^ab^ | 5.21±0.25^b^ | 1.03±0.25^b^ | 1.35±0.25 | 1.16±0.25^b^ | 0.64±0.25^b^ |
|  | GG (41) | 1.68±0.22^b^ | 5.57±0.22^b^ | 3.51±0.22^b^ | 3.69±0.22^b^ | 5.09±0.22^b^ | 1.16±0.22^b^ | 1.33±0.22 | 1.26±0.22^b^ | 0.60±0.22^b^ |
|  | *p* Value | 0.0006** | 0.0047** | <.0001** | 0.0233* | 0.0067** | 0.0018** | 0.3792 | 0.0124* | <.0001** |
| rs344498203 | CC (23) | 1.82±0.26^b^ | 5.74±0.26 | 3.90±0.25^b^ | 4.24±0.26 | 5.03±0.25^b^ | 0.98±0.25^b^ | 1.51±0.25 | 1.14±0.25^b^ | 0.70±0.25 |
|  | GC (22) | 2.29±0.28^ab^ | 6.09±0.28 | 4.06±0.28^ab^ | 3.83±0.28 | 5.72±0.28^ab^ | 1.92±0.31^a^ | 1.75±0.29 | 2.07±0.31^a^ | 1.24±0.30 |
|  | GG (75) | 2.43±0.21^a^ | 6.12±0.21 | 4.51±0.21^a^ | 4.03±0.21 | 5.66±0.21^a^ | 1.59±0.21^a^ | 1.37±0.21 | 1.50±0.22^ab^ | 1.18±0.21 |
|  | *p* Value | 0.0683 | 0.3296 | 0.0294* | 0.5046 | 0.0524 | 0.0356* | 0.3500 | 0.0489* | 0.1751 |
| rs323776551 | AA (41) | 2.51±0.23^a^ | 6.11±0.23^a^ | 4.66±0.24^a^ | 3.81±0.24 | 5.60±0.23^a^ | 1.55±0.23^ab^ | 1.24±0.24^a^ | 1.38±0.23^b^ | 1.32±0.24^a^ |
|  | CA (40) | 2.47±0.25^a^ | 6.33±0.24^a^ | 4.44±0.25^a^ | 4.26±0.25 | 5.87±0.24^a^ | 1.73±0.24^a^ | 1.62±0.24^b^ | 1.85±0.24^a^ | 1.19±0.24^a^ |
|  | CC (39) | 1.77±0.16^b^ | 5.66±0.22^b^ | 3.72±0.22^b^ | 4.06±0.21 | 5.05±0.22^b^ | 1.14±0.24^b^ | 1.54±0.22^b^ | 1.29±0.23^b^ | 0.65±0.24^b^ |
|  | *p* Value | 0.0067** | 0.0260* | 0.0009** | 0.1844 | 0.0039** | 0.0535 | 0.2167 | 0.0451* | 0.0399* |
| rs342083908 | AA (37) | 1.95±0.23^b^ | 5.63±0.22^b^ | 3.83±0.22^b^ | 4.11±0.22^ab^ | 4.95±0.22^b^ | 1.39±0.22 | 1.54±0.22 | 1.35±0.23 | 0.93±0.22 |
|  | GA (40) | 2.41±0.24^ab^ | 6.38±0.24^a^ | 4.45±0.24^a^ | 4.27±0.24^a^ | 5.94±0.24^a^ | 1.58±0.24 | 1.59±0.24 | 1.74±0.23 | 1.14±0.24 |
|  | GG (59) | 2.45±0.21^a^ | 6.23±0.21^a^ | 4.58±0.21^a^ | 3.79±0.21^b^ | 5.82±0.21^a^ | 1.54±0.21 | 1.39±0.21 | 1.61±0.21 | 1.30±0.21 |
|  | *p* Value | 0.0758 | 0.0053** | 0.0058** | 0.0775 | <.0001** | 0.7300 | 0.5982 | 0.2811 | 0.2857 |
| rs333053350 | GG (83) | 2.49±0.20^a^ | 6.23±0.20^a^ | 4.60±0.20^a^ | 4.20±0.20 | 5.69±0.20^a^ | 1.61±0.20^a^ | 1.49±0.20 | 1.66±0.20^a^ | 1.18±0.20^a^ |
|  | GT (63) | 2.00±0.19^b^ | 5.79±0.19^b^ | 3.90±0.19^b^ | 3.96±0.19 | 5.26±0.19^b^ | 1.39±0.19^a^ | 1.48±0.19 | 1.42±0.19^ab^ | 1.07±0.19^a^ |
|  | TT (8) | 0.44±0.41^c^ | 5.12±0.41^b^ | 1.88±0.41^c^ | 3.45±0.41 | 4.71±0.41^b^ | 0.14±0.41^b^ | 0.92±0.41 | 0.67±0.41^b^ | 0.06±0.41^b^ |
|  | *p* Value | <.0001** | 0.0021** | <.0001** | 0.1159 | 0.0060** | 0.0012** | 0.3056 | 0.0234* | 0.0069 |
| rs342210686 | AA (29) | 1.73±0.23^b^ | 5.80±0.23^b^ | 3.73±0.23^b^ | 4.09±0.23 | 5.27±0.23^b^ | 1.05±0.23^b^ | 1.39±0.23^b^ | 1.21±0.23^b^ | 0.76±0.23^b^ |
|  | GA (68) | 2.31±0.21^a^ | 5.83±0.21^b^ | 4.13±0.21^b^ | 3.89±0.21 | 5.30±0.21^b^ | 1.74±0.21^a^ | 1.53±0.21^b^ | 1.63±0.21^a^ | 1.31±0.21^a^ |
|  | GG (57) | 2.60±0.22^a^ | 6.38±0.22^a^ | 4.81±0.22^a^ | 4.27±0.22 | 5.85±0.22^a^ | 1.55±0.22^ab^ | 1.50±0.22^a^ | 1.67±0.22^a^ | 1.24±0.22^ab^ |
|  | *p* Value | 0.0022** | 0.0081** | <.0001** | 0.1660 | 0.0072** | 0.0196** | 0.8527* | 0.1524* | 0.0570 |
| rs328435752 | AA (86) | 2.44±0.20^a^ | 6.19±0.20^a^ | 4.55±0.20^a^ | 4.17±0.20 | 5.66±0.20^a^ | 1.56±0.20^a^ | 1.47±0.20 | 1.62±0.20^a^ | 1.15±0.20 |
|  | GA (60) | 2.05±0.19^b^ | 5.82±0.19^b^ | 3.94±0.19^b^ | 3.98±0.19 | 5.28±0.19^b^ | 1.43±0.19^a^ | 1.51±0.19 | 1.45±0.19^a^ | 1.10±0.19 |
|  | GG (8) | 0.42±0.42^c^ | 5.11±0.41^b^ | 1.86±0.41^c^ | 3.45±0.41 | 4.71±0.41^b^ | 0.12±0.41^b^ | 0.91±0.41 | 0.65±0.41^b^ | 0.07±0.41 |
|  | *p* Value | <.0001** | 0.0045** | <.0001** | 0.1591 | 0.0119* | 0.0018** | 0.2941 | 0.0384* | 0.0080** |
| rs787973778 | AA (86) | 2.44±0.20^a^ | 6.19±0.20^a^ | 4.55±0.20^a^ | 4.17±0.20 | 5.66±0.20^a^ | 1.56±0.20^a^ | 1.47±0.20 | 1.62±0.20^a^ | 1.15±0.20 |
|  | CA (60) | 2.05±0.19^b^ | 5.82±0.19^b^ | 3.94±0.19^b^ | 3.98±0.19 | 5.28±0.19^b^ | 1.43±0.19^a^ | 1.52±0.19 | 1.45±0.19^a^ | 1.10±0.19 |
|  | CC (8) | 0.42±0.42c | 5.11±0.41^b^ | 1.86±0.41^c^ | 3.45±0.41 | 4.71±0.41^b^ | 0.12±0.41^b^ | 0.91±0.40 | 0.65±0.41^b^ | 0.07±0.41 |
|  | *p* Value | <.0001** | 0.0045* | <.0001** | 0.1591 | 0.0119* | 0.0018** | 0.2941 | 0.0384* | 0.0080** |
| 24 h after mixing | | | | | | | | | | |
| rs339198696 | AA (52) | 2.87±0.23^b^ | 6.58±0.23^b^ | 4.85±0.22^b^ | 4.53±0.22^b^ | 6.16±0.24^b^ | 2.13±0.22^b^ | 2.39±0.23 | 2.05±0.23^b^ | 1.93±0.22^b^ |
|  | CA (24) | 2.95±0.27^b^ | 6.85±0.27^ab^ | 4.92±0.27^b^ | 5.02±0.26^ab^ | 6.54±0.28^ab^ | 2.26±0.27^ab^ | 2.58±0.27 | 2.37±0.27^ab^ | 1.89±0.27^b^ |
|  | CC (77) | 3.52±0.19^a^ | 7.10±0.19^a^ | 5.54±0.19^a^ | 5.09±0.19^a^ | 6.64±0.19^a^ | 2.73±0.19^a^ | 2.76±0.19 | 2.60±0.19^a^ | 2.50±0.19^a^ |
|  | *p* Value | 0.0017** | 0.0281* | 0.0008** | 0.0117* | 0.0518 | 0.0051** | 0.1578 | 0.0171* | 0.0035** |
| rs344700648 | AA (78) | 3.44±0.19^a^ | 7.07±0.19^a^ | 5.40±0.19^a^ | 5.01±0.19 | 6.63±0.19^a^ | 2.71±0.19^a^ | 2.69±0.19 | 2.64±0.19^a^ | 2.47±0.19^a^ |
|  | AT (32) | 3.27±0.25^ab^ | 7.03±0.25^ab^ | 5.43±0.25^a^ | 4.75±0.25 | 6.73±0.25^a^ | 2.32±0.25^ab^ | 2.75±0.26 | 2.10±0.25^b^ | 2.24±0.25^ab^ |
|  | TT (40) | 2.84±0.23^b^ | 6.64±0.23^b^ | 4.80±0.24^b^ | 4.90±0.24 | 6.14±0.23^b^ | 2.10±0.23^b^ | 2.45±0.24 | 2.06±0.23^b^ | 1.73±0.23^b^ |
|  | *p* Value | 0.0146* | 0.1089 | 0.0102* | 0.4570 | 0.0321* | 0.0087** | 0.3978 | 0.0053** | 0.0015** |
| rs335052970 | AA (76) | 3.68±0.20^a^ | 7.21±0.20^a^ | 5.72±0.20^a^ | 5.12±0.20^a^ | 6.74±0.20^a^ | 2.85±0.20^a^ | 2.81±0.20 | 2.71±0.20^a^ | 2.69±0.20^a^ |
|  | GA (38) | 3.20±0.24^b^ | 6.93±0.24^ab^ | 5.32±0.23^a^ | 5.00±0.24^ab^ | 6.50±0.24^ab^ | 2.26±0.24^b^ | 2.44±0.24 | 2.22±0.24^b^ | 1.87±0.24^b^ |
|  | GG (41) | 2.66±0.22^c^ | 6.62±0.22^b^ | 4.45±0.23^b^ | 4.71±0.22^b^ | 6.23±0.22^b^ | 2.14±0.22^b^ | 2.54±0.22 | 2.19±0.22^b^ | 1.79±0.22^b^ |
|  | *p* Value | <.0001** | 0.0173* | <.0001** | 0.1388 | 0.0534 | 0.0011** | 0.1596 | 0.0164* | <.0001** |
| rs344498203 | CC (23) | 3.01±0.26 | 6.73±0.26 | 5.13±0.26 | 5.07±0.25 | 6.14±0.25 | 2.08±0.26^b^ | 2.55±0.25 | 2.09±0.26^b^ | 2.20±0.26 |
|  | GC (22) | 3.40±0.29 | 7.08±0.29 | 5.15±0.29 | 4.73±0.29 | 6.86±0.29 | 2.96±0.29^a^ | 2.72±0.28 | 2.89±0.29^a^ | 2.21±0.29 |
|  | GG (75) | 3.39±0.22 | 7.03±0.22 | 5.41±0.22 | 5.04±0.22 | 6.61±0.22 | 2.61±0.22^a^ | 2.78±0.21 | 2.47±0.22^ab^ | 2.25±0.22 |
|  | *p* Value | 0.3490 | 0.4858 | 0.3971 | 0.4869 | 0.1104 | 0.0471* | 0.6698 | 0.0918 | 0.9690 |
| rs323776551 | AA (41) | 3.41±0.23 | 6.99±0.23 | 5.46±0.23 | 4.93±0.24 | 6.53±0.23^ab^ | 2.58±0.23^ab^ | 2.96±0.24 | 2.28±0.23^b^ | 2.36±0.23 |
|  | CA (40) | 3.46±0.24 | 7.19±0.24 | 5.34±0.24 | 5.01±0.24 | 6.87±0.24^a^ | 2.81±0.24^a^ | 2.67±0.24 | 2.74±0.24^a^ | 2.28±0.24 |
|  | CC (39) | 3.03±0.23 | 6.73±0.23 | 5.08±0.23 | 5.02±0.22 | 6.18±0.23^b^ | 2.19±0.23^b^ | 2.55±0.22 | 2.26±0.22^ab^ | 2.09±0.23 |
|  | *p* Value | 0.1770 | 0.1848 | 0.3012 | 0.9113 | 0.0231* | 0.0514 | 0.2095 | 0.0784 | 0.5670 |
| rs342083908 | AA (37) | 3.21±0.22 | 6.72±0.22 | 5.20±0.22 | 4.99±0.22 | 6.14±0.22^b^ | 2.47±0.22 | 2.48±0.22 | 2.43±0.23 | 2.19±0.22 |
|  | GA (40) | 3.36±0.24 | 7.22±0.24 | 5.34±0.24 | 4.96±0.24 | 6.94±0.24^a^ | 2.59±0.24 | 2.66±0.24 | 2.59±0.23 | 2.22±0.24 |
|  | GG (59) | 3.41±0.21 | 7.13±0.21 | 5.46±0.21 | 4.89±0.21 | 6.77±0.21^a^ | 2.58±0.21 | 2.87±0.22 | 2.47±0.21 | 2.43±0.21 |
|  | *p* Value | 0.6730 | 0.0954 | 0.5138 | 0.8859 | 0.0036** | 0.8559 | 0.2036 | 0.7596 | 0.4418 |
| rs333053350 | GG (83) | 3.46±0.20^a^ | 7.13±0.20^a^ | 5.56±0.20^a^ | 5.07±0.20 | 6.68±0.20^a^ | 2.57±0.20^a^ | 2.73±0.20 | 2.51±0.20^a^ | 2.30±0.20 |
|  | GT (63) | 3.23±0.19^a^ | 6.84±0.19^ab^ | 5.13±0.19^b^ | 4.88±0.19 | 6.41±0.19^a^ | 2.56±0.20^a^ | 2.62±0.19 | 2.45±0.19^a^ | 2.34±0.19 |
|  | TT (8) | 2.04±0.40^b^ | 6.23±0.40^b^ | 4.20±0.40^c^ | 4.81±0.40 | 5.54±0.40^b^ | 1.02±0.40^b^ | 2.55±0.40 | 1.47±0.40^b^ | 2.05±0.40 |
|  | *p* Value | 0.0008** | 0.0270* | 0.0005** | 0.4798 | 0.0078** | 0.0003** | 0.7753 | 0.0258* | 0.7651 |
| rs342210686 | AA (29) | 2.99±0.23^b^ | 6.73±0.23 | 5.05±0.23^b^ | 4.92±0.23 | 6.24±0.23 | 2.13±0.23^b^ | 2.50±0.23 | 2.08±0.23^b^ | 2.10±0.23 |
|  | GA (68) | 3.41±0.21^ab^ | 6.98±0.21 | 5.25±0.21^b^ | 4.88±0.21 | 6.58±0.21 | 2.82±0.21^a^ | 2.62±0.21 | 2.67±0.21^a^ | 2.44±0.21 |
|  | GG (57) | 3.54±0.23^a^ | 7.21±0.22 | 5.70±0.23^a^ | 5.15±0.23 | 6.76±0.23 | 2.56±0.23^ab^ | 2.89±0.23 | 2.50±0.23^ab^ | 2.34±0.23 |
|  | *p* Value | 0.0689 | 0.1357 | 0.0154* | 0.3588 | 0.1009 | 0.0176* | 0.1971 | 0.0464* | 0.3380 |
| rs328435752 | AA (86) | 3.45±0.20^a^ | 7.10±0.20^a^ | 5.54±0.20^a^ | 5.07±0.20 | 6.65±0.20^a^ | 2.56±0.20^a^ | 2.73±0.20 | 2.48±0.20^a^ | 2.33±0.20 |
|  | GA (60) | 3.23±0.20^a^ | 6.86±0.20^ab^ | 5.13±0.20^b^ | 4.87±0.20 | 6.44±0.20^a^ | 2.57±0.20^a^ | 2.61±0.20 | 2.47±0.20^a^ | 2.30±0.20 |
|  | GG (8) | 2.03±0.40^b^ | 6.22±0.40^b^ | 4.19±0.40^c^ | 4.81±0.40 | 5.54±0.40^b^ | 1.02±0.40^b^ | 2.55±0.40 | 1.47±0.40^b^ | 2.06±0.40 |
|  | *p* Value | 0.0010** | 0.0385* | 0.0006** | 0.4524 | 0.0123* | 0.0003** | 0.7565 | 0.0276* | 0.7801 |
| rs787973778 | AA (86) | 3.45±0.20^a^ | 7.10±0.20^a^ | 5.54±0.20^a^ | 5.07±0.20 | 6.65±0.20^a^ | 2.56±0.20^a^ | 2.73±0.20 | 2.48±0.20^a^ | 2.33±0.20 |
|  | GA (60) | 3.23±0.20^a^ | 6.86±0.20^ab^ | 5.13±0.20^b^ | 4.87±0.20 | 6.44±0.20^a^ | 2.57±0.20^a^ | 2.61±0.20 | 2.47±0.20^a^ | 2.30±0.20 |
|  | GG (8) | 2.03±0.40^b^ | 6.22±0.40^b^ | 4.19±0.40^c^ | 4.81±0.40 | 5.54±0.40^b^ | 1.02±0.40^b^ | 2.55±0.40 | 1.47±0.40^b^ | 2.06±0.40 |
|  | *p* Value | 0.0010** | 0.0385* | 0.0006** | 0.4524 | 0.0123* | 0.0003** | 0.7565 | 0.0276* | 0.7801 |
| 48 h after mixing | | | | | | | | | | |
| rs339198696 | AA (52) | 3.11±0.22^b^ | 6.70±0.22^b^ | 5.06±0.22^b^ | 4.72±0.22^b^ | 6.23±0.22^b^ | 2.39±0.22^b^ | 2.57±0.23^b^ | 2.16±0.22^b^ | 2.19±0.22^b^ |
|  | CA (24) | 3.02±0.27^b^ | 6.89±0.27^ab^ | 5.01±0.27^b^ | 5.08±0.27^ab^ | 6.59±0.28^ab^ | 2.31±0.27^b^ | 2.65±0.27^b^ | 2.41±0.27^ab^ | 1.98±0.27^b^ |
|  | CC (77) | 3.63±0.19^a^ | 7.20±0.19^a^ | 5.63±0.19^a^ | 5.25±0.19^a^ | 6.71±0.19^a^ | 2.86±0.19^a^ | 2.89±0.19^a^ | 2.70±0.19^a^ | 2.60±0.19^a^ |
|  | *p* Value | 0.0061** | 0.0349* | 0.0033** | 0.0211* | 0.0472* | 0.0162* | 0.2232 | 0.0184* | 0.0148* |
| rs344700648 | AA (78) | 3.55±0.19^a^ | 7.15±0.19 | 5.50±0.19^a^ | 5.15±0.19 | 6.69±0.19^a^ | 2.83±0.19^a^ | 2.83±0.19 | 2.69±0.19^a^ | 2.57±0.19^a^ |
|  | AT (32) | 3.42±0.25^ab^ | 7.12±0.25 | 5.55±0.25^a^ | 4.98±0.25 | 6.78±0.25^a^ | 2.53±0.25^ab^ | 2.85±0.26 | 2.24±0.25^a^ | 2.42±0.25^a^ |
|  | TT (40) | 3.04±0.24^b^ | 6.78±0.23 | 4.98±0.24^b^ | 5.05±0.24 | 6.26±0.23^b^ | 2.33±0.24^b^ | 2.58±0.24 | 2.28±0.24^b^ | 1.91±0.23^b^ |
|  | *p* Value | 0.0423* | 0.1728 | 0.0256* | 0.7136 | 0.0644 | 0.0425* | 0.4222 | 0.0462* | 0.0052** |
| rs335052970 | AA (76) | 3.76±0.20^a^ | 7.29±0.20^a^ | 5.78±0.20^a^ | 5.27±0.20^a^ | 6.80±0.20^a^ | 2.97±0.20^a^ | 2.93±0.20 | 2.80±0.20^a^ | 2.74±0.20^a^ |
|  | GA (38) | 3.26±0.23^b^ | 6.97±0.23^ab^ | 5.36±0.23^b^ | 5.11±0.23^ab^ | 6.54±0.23^ab^ | 2.38±0.23^b^ | 2.57±0.23 | 2.33±0.23^b^ | 2.01±0.23^b^ |
|  | GG (41) | 2.95±0.22^b^ | 6.74±0.22^b^ | 4.76±0.22^c^ | 4.84±0.22^b^ | 6.33±0.22^b^ | 2.39±0.22^b^ | 2.69±0.22 | 2.27±0.22^b^ | 2.06±0.22^b^ |
|  | *p* Value | 0.0004** | 0.0270* | <.0001** | 0.1044 | 0.0724 | 0.0044** | 0.1860 | 0.0154* | 0.0003** |
| rs344498203 | CC (23) | 3.27±0.25 | 6.90±0.25 | 5.35±0.25 | 5.17±0.26 | 6.32±0.25^b^ | 2.40±0.25^b^ | 2.72±0.25 | 2.29±0.25 | 2.37±0.25 |
|  | GC (22) | 3.51±0.29 | 7.13±0.29 | 5.32±0.29 | 4.98±0.29 | 6.77±0.29^ab^ | 2.96±0.29^a^ | 2.81±0.29 | 2.86±0.29 | 2.30±0.29 |
|  | GG (75) | 3.47±0.21 | 7.10±0.21 | 5.47±0.21 | 5.20±0.22 | 6.65±0.21^a^ | 2.71±0.21^a^ | 2.90±0.21 | 2.53±0.22 | 2.37±0.21 |
|  | *p* Value | 0.7148 | 0.7239 | 0.7914 | 0.7110 | 0.3775 | 0.2722 | 0.7485 | 0.2712 | 0.9613 |
| rs323776551 | AA (41) | 3.51±0.23 | 7.08±0.23 | 5.51±0.24 | 5.12±0.24 | 6.60±0.23^ab^ | 2.75±0.23^ab^ | 3.09±0.24 | 2.39±0.23 | 2.50±0.24 |
|  | CA (40) | 3.55±0.25 | 7.22±0.24 | 5.44±0.25 | 5.15±0.25 | 6.85±0.24^a^ | 2.90±0.24^a^ | 2.78±0.24 | 2.77±0.24 | 2.43±0.24 |
|  | CC (39) | 3.25±0.22 | 6.89±0.22 | 5.31±0.22 | 5.16±0.22 | 6.33±0.22^b^ | 2.40±0.22^b^ | 2.69±0.22 | 2.39±0.22 | 2.22±0.22 |
|  | *p* Value | 0.4316 | 0.4082 | 0.7104 | 0.9871 | 0.1067 | 0.1281 | 0.1879 | 0.1899 | 0.5140 |
| rs342083908 | AA (37) | 3.37±0.23^b^ | 6.86±0.22 | 5.35±0.22 | 5.12±0.22 | 6.28±0.22^b^ | 2.65±0.22 | 2.62±0.22 | 2.52±0.23 | 2.35±0.22 |
|  | GA (40) | 3.49±0.24^ab^ | 7.28±0.24 | 5.49±0.24 | 5.11±0.24 | 6.95±0.24^a^ | 2.71±0.24 | 2.76±0.24 | 2.70±0.23 | 2.33±0.24 |
|  | GG (59) | 3.54±0.21^a^ | 7.21±0.21 | 5.55±0.21 | 5.09±0.21 | 6.80±0.21^a^ | 2.78±0.21 | 3.04±0.22 | 2.56±0.21 | 2.58±0.21 |
|  | *p* Value | 0.7617 | 0.1772 | 0.6713 | 0.9859 | 0.0185* | 0.8484 | 0.1475 | 0.7245 | 0.4152 |
| rs333053350 | GG (83) | 3.60±0.20^a^ | 7.23±0.20^a^ | 5.67±0.20^a^ | 5.24±0.20 | 6.76±0.20^a^ | 2.76±0.20^a^ | 2.89±0.20 | 2.61±0.20^a^ | 2.46±0.20^a^ |
|  | GT (63) | 3.34±0.19^ab^ | 6.92±0.19^b^ | 5.24±0.19^b^ | 5.02±0.19 | 6.46±0.19^b^ | 2.69±0.19^a^ | 2.71±0.19 | 2.53±0.19^ab^ | 2.43±0.19^a^ |
|  | TT (8) | 2.80±0.40^b^ | 6.52±0.40^b^ | 4.91±0.40^c^ | 4.98±0.40 | 5.68±0.40^b^ | 1.88±0.40^b^ | 2.74±0.40 | 1.86±0.40^b^ | 2.35±0.40^b^ |
|  | *p* Value | 0.0597 | 0.0585 | 0.0161* | 0.4119 | 0.0088** | 0.0694 | 0.5823 | 0.1376 | 0.9580 |
| rs342210686 | AA (29) | 3.21±0.23^b^ | 6.86±0.23^b^ | 5.22±0.23^b^ | 5.03±0.23 | 6.36±0.23 | 2.43±0.23 | 2.64±0.23 | 2.22±0.23^b^ | 2.29±0.23 |
|  | GA (68) | 3.49±0.21^a^ | 7.06±0.21^b^ | 5.35±0.21^b^ | 5.07±0.21 | 6.61±0.21 | 2.89±0.21 | 2.76±0.21 | 2.72±0.21^a^ | 2.53±0.21 |
|  | GG (57) | 3.64±0.21^a^ | 7.29±0.21^a^ | 5.77±0.22^a^ | 5.27±0.22 | 6.86±0.22 | 2.73±0.21 | 3.01±0.21 | 2.63±0.21^ab^ | 2.48±0.21 |
|  | *p* Value | 0.1998 | 0.1662 | 0.0406* | 0.4902 | 0.1000 | 0.1473 | 0.2181 | 0.1016 | 0.5591 |
| rs328435752 | AA (86) | 3.58±0.20^a^ | 7.21±0.20^a^ | 5.64±0.20^a^ | 5.23±0.20 | 6.74±0.20^a^ | 2.75±0.20^a^ | 2.89±0.20 | 2.59±0.20 | 2.49±0.20 |
|  | CA (60) | 3.35±0.20^b^ | 6.94±0.20^b^ | 5.25±0.20^b^ | 5.01±0.20 | 6.48±0.20^a^ | 2.70±0.20^a^ | 2.71±0.20 | 2.55±0.20 | 2.40±0.20 |
|  | CC (8) | 2.79±0.40c | 6.51±0.40^b^ | 4.90±0.40^ab^ | 4.98±0.40 | 5.67±0.40^b^ | 1.87±0.40^b^ | 2.75±0.40 | 1.85±0.40 | 2.36±0.40 |
|  | *p* Value | 0.0738 | 0.0832 | 0.0252* | 0.4032 | 0.0136* | 0.0722 | 0.5791 | 0.1530 | 0.8568 |
| rs787973778 | AA (86) | 3.58±0.20^a^ | 7.21±0.20^a^ | 5.64±0.20^a^ | 5.23±0.20 | 6.74±0.20^a^ | 2.75±0.20^a^ | 2.89±0.20 | 2.59±0.20 | 2.49±0.20 |
|  | CA (60) | 3.35±0.20^ab^ | 6.94±0.20^b^ | 5.25±0.20^b^ | 5.01±0.20 | 6.48±0.20^a^ | 2.70±0.20^a^ | 2.71±0.20 | 2.55±0.20 | 2.40±0.20 |
|  | CC (8) | 2.79±0.40^b^ | 6.51±0.40^b^ | 4.90±0.40^ab^ | 4.98±0.40 | 5.67±0.40^b^ | 1.87±0.40^b^ | 2.75±0.40 | 1.85±0.40 | 2.36±0.40 |
|  | *p* Value | 0.0738 | 0.0832 | 0.0252* | 0.4032 | 0.0136* | 0.0722 | 0.5791 | 0.1530 | 0.8568 |
| 72 h after mixing | | | | | | | | | | |
| rs339198696 | AA (52) | 3.20±0.22^b^ | 6.81±0.22^b^ | 5.11±0.22^b^ | 4.84±0.22^b^ | 6.30±0.22^b^ | 2.55±0.22^b^ | 3.02±0.23 | 2.20±0.22^b^ | 2.33±0.22^b^ |
|  | CA (24) | 3.05±0.27^b^ | 6.92±0.27^ab^ | 5.03±0.27^b^ | 5.08±0.27^ab^ | 6.61±0.28^ab^ | 2.34±0.27^b^ | 2.84±0.27 | 2.38±0.27^ab^ | 2.03±0.27^b^ |
|  | CC (77) | 3.67±0.19^a^ | 7.26±0.19^a^ | 5.65±0.19^a^ | 5.32±0.19^a^ | 6.75±0.19^a^ | 2.92±0.19^a^ | 3.25±0.19 | 2.76±0.19^a^ | 2.65±0.19^a^ |
|  | *p* Value | 0.0094** | 0.0547 | 0.0044** | 0.0457* | 0.0620 | 0.0284* | 0.1750 | 0.0108* | 0.0286* |
| rs344700648 | AA (78) | 3.60±0.19^a^ | 7.20±0.19 | 5.52±0.19^a^ | 5.21±0.19 | 6.72±0.19^a^ | 2.89±0.19^a^ | 3.19±0.19 | 2.70±0.19^a^ | 2.63±0.19^a^ |
|  | AT (32) | 3.46±0.25^ab^ | 7.17±0.25 | 5.56±0.25^a^ | 5.10±0.25 | 6.80±0.25^a^ | 2.61±0.25^ab^ | 3.20±0.26 | 2.46±0.25^a^ | 2.52±0.25^a^ |
|  | TT (40) | 3.12±0.24^b^ | 6.85±0.23 | 5.00±0.24^b^ | 5.11±0.24 | 6.30±0.23^b^ | 2.44±0.24^b^ | 2.98±0.24 | 2.30±0.24^b^ | 1.96±0.23^b^ |
|  | *p* Value | 0.0648 | 0.2200 | 0.0295* | 0.8146 | 0.0772 | 0.0646 | 0.5732 | 0.1282 | 0.0049** |
| rs335052970 | AA (76) | 3.79±0.20^a^ | 7.32±0.20^a^ | 5.80±0.20^a^ | 5.31±0.20 | 6.83±0.20^a^ | 3.00±0.20^a^ | 3.20±0.20 | 2.80±0.20^a^ | 2.77±0.20^a^ |
|  | GA (38) | 3.33±0.23^b^ | 7.03±0.23^ab^ | 5.39±0.23^b^ | 5.16±0.23 | 6.59±0.23^ab^ | 2.48±0.23^b^ | 3.04±0.23 | 2.33±0.23^ab^ | 2.11±0.23^b^ |
|  | GG (41) | 3.07±0.22^b^ | 6.84±0.22^b^ | 4.79±0.22^c^ | 4.99±0.22 | 6.36±0.22^b^ | 2.56±0.22^b^ | 3.15±0.22 | 2.27±0.22^b^ | 2.21±0.22^b^ |
|  | *p* Value | 0.0018** | 0.0596 | <.0001** | 0.2800 | 0.0785 | 0.0206** | 0.7390 | 0.0439* | 0.0022** |
| rs344498203 | CC (23) | 3.34±0.25 | 6.99±0.25 | 5.40±0.25 | 5.27±0.26 | 6.38±0.25 | 2.50±0.25 | 3.12±0.25 | 2.34±0.25 | 2.46±0.25 |
|  | GC (22) | 3.53±0.29 | 7.16±0.29 | 5.33±0.29 | 4.99±0.29 | 6.81±0.29 | 3.01±0.29 | 2.88±0.29 | 2.90±0.29 | 2.34±0.29 |
|  | GG (75) | 3.53±0.21 | 7.17±0.21 | 5.49±0.21 | 5.28±0.22 | 6.70±0.21 | 2.80±0.21 | 3.31±0.21 | 2.57±0.22 | 2.44±0.21 |
|  | *p* Value | 0.7668 | 0.7787 | 0.7894 | 0.5359 | 0.3933 | 0.3216 | 0.2153 | 0.2868 | 0.9258 |
| rs323776551 | AA (41) | 3.58±0.23 | 7.17±0.23 | 5.53±0.24 | 5.23±0.24 | 6.66±0.23^ab^ | 2.86±0.23^ab^ | 3.59±0.24^a^ | 2.46±0.23 | 2.59±0.24 |
|  | CA (40) | 3.59±0.25 | 7.27±0.24 | 5.45±0.25 | 5.19±0.25 | 6.90±0.24^a^ | 2.95±0.24^a^ | 2.97±0.24^b^ | 2.82±0.24 | 2.49±0.24 |
|  | CC (39) | 3.31±0.22 | 6.95±0.22 | 5.34±0.22 | 5.24±0.22 | 6.37±0.22^b^ | 2.50±0.22^b^ | 2.99±0.22^b^ | 2.43±0.22 | 2.27±0.22 |
|  | *p* Value | 0.4569 | 0.4251 | 0.7422 | 0.9769 | 0.1034 | 0.1614 | 0.0141* | 0.1901 | 0.4442 |
| rs342083908 | AA (37) | 3.43±0.22 | 6.93±0.22 | 5.38±0.22 | 5.22±0.22 | 6.33±0.22^b^ | 2.73±0.22 | 3.06±0.22 | 2.56±0.22 | 2.42±0.22 |
|  | GA (40) | 3.52±0.24 | 7.32±0.24 | 5.50±0.24 | 5.14±0.24 | 6.98±0.24^a^ | 2.75±0.24 | 2.89±0.24 | 2.74±0.23 | 2.39±0.24 |
|  | GG (59) | 3.60±0.21 | 7.27±0.21 | 5.57±0.21 | 5.17±0.21 | 6.84±0.21^a^ | 2.86±0.21 | 3.48±0.22 | 2.60±0.21 | 2.64±0.21 |
|  | *p* Value | 0.7575 | 0.2152 | 0.6908 | 0.9514 | 0.0228* | 0.7975 | 0.0264* | 0.7371 | 0.4193 |
| rs333053350 | GG (83) | 3.65±0.20^a^ | 7.28±0.20 | 5.69±0.20^a^ | 5.31±0.21 | 6.79±0.20^a^ | 2.83±0.20^a^ | 3.29±0.20 | 2.68±0.20^a^ | 2.52±0.20^a^ |
|  | GT (63) | 3.40±0.19^ab^ | 6.98±0.19 | 5.27±0.19^b^ | 5.08±0.19 | 6.50±0.19^a^ | 2.77±0.19^a^ | 3.05±0.20 | 2.55±0.19^ab^ | 2.49±0.19^a^ |
|  | TT (8) | 2.87±0.40^b^ | 6.61±0.40 | 4.94±0.40^c^ | 5.16±0.40 | 5.71±0.40^b^ | 1.99±0.40^b^ | 2.86±0.40 | 1.90±0.40^b^ | 2.46±0.40^b^ |
|  | *p* Value | 0.0746 | 0.0741 | 0.0182* | 0.3850 | 0.0092** | 0.0859 | 0.2509 | 0.1127 | 0.9703 |
| rs342210686 | AA (29) | 3.27±0.23^b^ | 6.92±0.23^b^ | 5.25±0.23^b^ | 5.11±0.23 | 6.39±0.23^b^ | 2.53±0.23 | 2.99±0.23^b^ | 2.25±0.23^b^ | 2.36±0.23 |
|  | GA (68) | 3.53±0.21^a^ | 7.10±0.21^b^ | 5.38±0.21^b^ | 5.11±0.21 | 6.63±0.21^ab^ | 2.93±0.21 | 2.94±0.21^b^ | 2.74±0.21^a^ | 2.56±0.21 |
|  | GG (57) | 3.70±0.21^a^ | 7.37±0.21^a^ | 5.79±0.22^a^ | 5.38±0.22 | 6.90±0.22^a^ | 2.83±0.21 | 3.50±0.22^a^ | 2.73±0.21^a^ | 2.56±0.21 |
|  | *p* Value | 0.2055 | 0.1405 | 0.0424* | 0.3291 | 0.0963 | 0.2442 | 0.0081** | 0.0805 | 0.6519 |
| rs328435752 | AA (86) | 3.63±0.20^a^ | 7.26±0.20 | 5.67±0.20^a^ | 5.32±0.20 | 6.77±0.20^a^ | 2.83±0.20^a^ | 2.34±0.20^a^ | 2.65±0.20^a^ | 2.55±0.20 |
|  | CA (60) | 3.40±0.20^bc^ | 6.99±0.20 | 5.27±0.20^b^ | 5.06±0.20 | 6.52±0.20^a^ | 2.77±0.20^a^ | 2.98±0.20^a^ | 2.57±0.20^ab^ | 2.45±0.20 |
|  | CC (8) | 2.87±0.40^b^ | 6.61±0.40 | 4.93±0.40^ab^ | 5.16±0.40 | 5.71±0.40^b^ | 1.99±0.40^b^ | 2.88±0.40^b^ | 1.89±0.40^b^ | 2.46±0.40 |
|  | *p* Value | 0.0844 | 0.0935 | 0.0279* | 0.3351 | 0.0141* | 0.0872 | 0.0984 | 0.1355 | 0.8363 |
| rs787973778 | AA (86) | 3.63±0.20^a^ | 7.26±0.20 | 5.67±0.20^a^ | 5.32±0.20 | 6.77±0.20^a^ | 2.83±0.20^a^ | 2.34±0.20^a^ | 2.65±0.20^a^ | 2.55±0.20 |
|  | CA (60) | 3.40±0.20^ab^ | 6.99±0.20 | 5.27±0.20^b^ | 5.06±0.20 | 6.52±0.20^a^ | 2.77±0.20^a^ | 2.98±0.20^a^ | 2.57±0.20^ab^ | 2.45±0.20 |
|  | CC (8) | 2.87±0.40^b^ | 6.61±0.40 | 4.93±0.40^ab^ | 5.16±0.40 | 5.71±0.40^b^ | 1.99±0.40^b^ | 2.88±0.40^b^ | 1.89±0.40^b^ | 2.46±0.40 |
|  | *p* Value | 0.0844 | 0.0935 | 0.0279* | 0.3351 | 0.0141* | 0.0872 | 0.0984 | 0.1355 | 0.8363 |

Note: *P*-value shows the significance for genetic effects among the SNPs; ^abcd^ within the same column with different superscripts means significant differences, and the same superscript indicates no significant differences.
