# Supplementary material for: A Single-Nucleotide Polymorphism in the Promoter of Porcine ARHGAP24 Gene Regulates Aggressive Behavior of Weaned Pigs After Mixing by Affecting the Binding of Transcription Factor p53
Source: Front Cell Dev Biol. 2022 Apr 1;10:839583. doi: 10.3389/fcell.2022.839583 (PMC9010951; doi:10.3389/fcell.2022.839583)
Supplement: Supplementary file 2 [file Table6.DOC]

# Supplementary Tables

**Supplementary Table 6.** The change of transcription factor-binding sites caused by the SNPs in the core promoter region and 5’ UTR of the porcine ARHGAP24 gene predicted by a bioinformatics website.

| SNP | Predicted sequence | Transcription factor | Matched sequence | Score | *P*-value | Q-value |
| --- | --- | --- | --- | --- | --- | --- |
| rs339198696 | CAAACCAAAACCAAAAACCAAAAACAAAAAC | RUNX2 | AAACCAAAAACCAAAA | 12.9242 | 1.64E-05 | 0.000351 |
|  |  | RREB1 | ACCAAAACCAAAAACCAAAA | 8.06422 | 1.70E-05 | 0.000249 |
|  |  | IRF2 | CAAACCAAAACCAAAAACC | 7.37879 | 4.92E-05 | 0.00128 |
|  |  | IRF1 | AAACCAAAACCAAAAACC | 6.04545 | 5.48E-05 | 0.00153 |
|  | CAAACCAAAACCAAACACCAAAAACAAAAAC | RREB1 | AAACCAAACACCAAAAACAA | 12.7982 | 3.01E-06 | 7.21E-05 |
|  |  | RUNX2 | AACCAAACACCAAAAA | 13.8734 | 8.31E-06 | 0.000266 |
|  |  | CREBBP | AAACCAAAACCAAACACCA | 12.1711 | 1.48E-05 | 0.000386 |
|  |  | FOXM1 | AAAACCAAACACC | 11.4706 | 4.57E-05 | 0.00173 |
| rs344700648 | AAATCACTAAGTTGGTGTTCCAGGTAACCGG | GLI1 | AGTTGGTGTTCC | 13.0612 | 1.79E-05 | 0.000714 |
|  |  | KDM5B | GTGTTCCAGGTAACCG | 9.11842 | 4.95E-05 | 0.00158 |
|  |  | GLI3 | GTTGGTGTTCC | 11.1531 | 5.09E-05 | 0.00214 |
|  | AAATCACTAAGTTGGAGTTCCAGGTAACCGG | POLR3A | CTAAGTTGGAGTTCCAG | 9.4 | 6.04E-05 | 0.00181 |
|  |  | GLI1 | AGTTGGAGTTCC | 10.4898 | 7.35E-05 | 0.00294 |
|  |  | STAT1 | GGAGTTCCAGGTAACC | 9.5 | 7.71E-05 | 0.00247 |
| rs335052970 | GGTACTGTCCTGTCCGGGTTTGAAAGAGACC | FOXM1 | TGTCCGGGTTTGAAA | 13.7639 | 7.12E-06 | 0.000242 |
|  |  | E2F4 | CTGTCCGGGTTTGAAA | 11.6667 | 4.24E-05 | 0.00136 |
|  |  | ZNF524 | TGTCCGGGTTTGAA | 9.85714 | 7.17E-05 | 0.00258 |
|  | GGTACTGTCCTGTCCAGGTTTGAAAGAGACC | FOXM1 | TGTCCAGGTTTGAAA | 13.8333 | 6.39E-06 | 0.000217 |
|  |  | E2F4 | CTGTCCAGGTTTGAAA | 11.4028 | 5.04E-05 | 0.00161 |
|  |  | TP53 | CCTGTCCAG | 11.3816 | 5.93E-05 | 0.00273 |
| rs333053350 | GATCCGGGACCTGGAGAATCCAAATCTTCAA | ETS1 | ATCCGGGACCTGGAGA | 9.01316 | 5.96E-05 | 0.00191 |
|  |  | ESR1 | TCTCCAGGTCCC | 10.5 | 6.12E-05 | 0.00245 |
|  |  | MECP2 | AGGTCCCGGATC | 9.10526 | 9.08E-05 | 0.00363 |
|  | GATCCGGGACCTGGATAATCCAAATCTTCAA | PITX3 | TGGATTATC | 11.8716 | 2.28E-05 | 0.00105 |
|  |  | MECP2 | CCGGGACCTGGATAA | 11.5102 | 7.16E-05 | 0.00243 |
|  |  | FOXL2 | TAATCCAAATCT | 11.5328 | 7.96E-05 | 0.00319 |
| rs342210686 | ACTTAACCCTGATCCGGGACCTGGAGAATCC | SPDEF | CCTGATCCGGGA | 12.0143 | 4.54E-05 | 0.00182 |
|  |  | GABPA | ATCCGGGACCTGGAGA | 10.9079 | 5.33E-05 | 0.0017 |
|  |  | ETS1 | ATCCGGGACCTGGAGA | 9.01316 | 5.96E-05 | 0.00191 |
|  | ACTTAACCCTGATCCAGGACCTGGAGAATCC | FOSL1 | CCCTGATCCAGGACCTGGAG | 8.3 | 3.34E-05 | 0.000802 |
|  |  | REST | CCAGGTCCTGGA | 11.6053 | 3.50E-05 | 0.0014 |
|  |  | TAL1 | TCCAGGACCTGGAGAA | 8.64474 | 3.66E-05 | 0.00117 |
| rs328435752 | AGTAAGTGAGAGGTGATCACTTAACCCTGAT | ZNF274 | GTGATCACCTCTCACT | 14.5352 | 4.67E-06 | 0.000149 |
|  |  | HMX3 | GGTTAAGTGAT | 10.24 | 5.49E-05 | 0.00231 |
|  |  | GATA1 | GTAAGTGAGAGGTGAT | 10.3465 | 8.75E-05 | 0.0028 |
|  | AGTAAGTGAGAGGTGGTCACTTAACCCTGAT | ZNF143 | GAGGTGGTCACTTAACCCTGAT | 10.6316 | 3.73E-05 | 0.000745 |
|  |  | GATA3 | TGGTCACTTAACCCTG | 10.3684 | 6.59E-05 | 0.00211 |
|  |  | ESR1 | TGGTCACTTAACCCTG | 10.7879 | 6.90E-05 | 0.00221 |
| rs787973778 | AAAACTTAAAAAAAAAAAAAAAAAACAAACC | IRF5 | AAAAAAAAAAAAAAAA | 27.1169 | 1.04E-09 | 1.11E-08 |
|  |  | POLR3A | AAAAAAAAAAAAAAAA | 19.5769 | 1.04E-09 | 1.11E-08 |
|  |  | ZNF143 | AAAAAAAAAAAAAAAAACAAA | 16.9744 | 2.03E-07 | 4.46E-06 |
|  | AAAACTTAAAAAAAACAAAAAAAAACAAACC | EZH2 | ACTTAAAAAAAACAAAAAAAAA | 11.9302 | 1.32E-05 | 0.000264 |
|  |  | NOTCH1 | AAAACAAAAAAAAACA | 11.9872 | 3.23E-05 | 0.00103 |
|  |  | SMAD1 | AAAAACAAAAAA | 11.9018 | 3.40E-05 | 0.00136 |

Note: underline type is the allele of SNPs.
