# Supplementary material for: A Single-Nucleotide Polymorphism in the Promoter of Porcine ARHGAP24 Gene Regulates Aggressive Behavior of Weaned Pigs After Mixing by Affecting the Binding of Transcription Factor p53
Source: Front Cell Dev Biol. 2022 Apr 1;10:839583. doi: 10.3389/fcell.2022.839583 (PMC9010951; doi:10.3389/fcell.2022.839583)
Supplement: Supplementary file 4 [file Table1.DOC]

Supplementary Material

**A Single Nucleotide Polymorphism in Promoter of Porcine *ARHGAP24* Gene Regulates Aggressive Behavior of Weaned Pigs after Mixing by Affecting the Binding of Transcription Factor p53**

# Supplementary Tables

**Table S1.** The primers information of SNP identification for porcine *ARHGAP24* gene.

| Primer | Primer sequence (5'-3') | Product Size (bp) | Usage |
| --- | --- | --- | --- |
| ARHGAP24-1 | F: 5’-ACATGAGATATTCTGGGGACAA-3’  R: 5’-CCTGTAGCCTGGTTTGGGAA-3’ | 626 | SNP identiﬁcation |
| ARHGAP24-2 | F: 5’-GGGGTGGGAGAATGTGTTTA-3’  R: 5’-CACTTTAAAGCAAAAGGGCATT-3’ | 153 | SNP identiﬁcation |
| ARHGAP24-3 | F: 5’-GTTCCTTTGGCTGTGACAT-3’  R: 5’-GGTACCCCACAGGCACTC-3’ | 242 | SNP identiﬁcation |
| ARHGAP24-4 | F: 5’-ATCACCAGGAAGAAAGAC-3’  R: 5’-AGAGAAATTCAAAGCATG-3’ | 439 | SNP identiﬁcation |
| ARHGAP24-5 | F: 5’-CGAGGGAAACCCGTGTTCAA-3’  R: 5’-AGACGGCTACAATGTGTGGG-3’ | 577 | SNP identiﬁcation |
| ARHGAP24-6 | F: 5’-TGGTAGCTTCCAAACCCTGG-3’  R: 5’-CATTCCCGCCATACCAAGGA-3’ | 476 | SNP identification |
| ARHGAP24-7 | F: 5’-GTTGGGAAATTGAGCAAA-3’  R: 5’-AGTGAAAGAATACACGGCA-3’ | 203 | SNP identiﬁcation |
| ARHGAP24-8 | F: 5’-TGAACAACAGCAGGCAAA-3’  R: 5’-TCAACAGGGAAGAACAATA-3’ | 146 | SNP identiﬁcation |
