# Supplementary material for: A Single-Nucleotide Polymorphism in the Promoter of Porcine ARHGAP24 Gene Regulates Aggressive Behavior of Weaned Pigs After Mixing by Affecting the Binding of Transcription Factor p53
Source: Front Cell Dev Biol. 2022 Apr 1;10:839583. doi: 10.3389/fcell.2022.839583 (PMC9010951; doi:10.3389/fcell.2022.839583)
Supplement: Supplementary file 5 [file Table5.DOC]

# Supplementary Tables

**Table S5.** Significant associations of haplotype block with aggressive behavioral traits in pigs (LSM ± SE).

| Haplotype  block | Haplotype combination (No.) | CAS | Duration of fights(s) | Duration of active attacks(s) | Duration of being bullied(s) | Duration of standoff(s) | Frequency of active attacks | Frequency of being bullied | Frequency of standoff | Win | |
| --- | --- | --- | --- | --- | --- | --- | --- | --- | --- | --- | --- |
| 2 h after mixing | | | | | | | | | | | |
| Block1 | H1H1 (55) | 2.53±0.23^a^ | 6.32±0.23^a^ | 4.72±0.23^a^ | 4.28±0.23^a^ | 5.80±0.23^a^ | 1.48±0.23^a^ | 1.51±0.23 | 1.64±0.23^a^ | 1.17±0.23^a^ | |
|  | H1H2 (41) | 2.03±0.24^b^ | 5.62±0.24^bc^ | 3.83±0.24^b^ | 3.71±0.24^b^ | 5.12±0.24^bc^ | 1.47±0.24^a^ | 1.46±0.24 | 1.45±0.24^ab^ | 1.19±0.24^a^ | |
|  | H1H3 (26) | 2.49±0.26^ab^ | 5.94±0.26^abc^ | 4.33±0.26^ab^ | 4.05±0.26^ab^ | 5.38±0.26^ab^ | 1.90±0.26^a^ | 1.52±0.26 | 1.76±0.26^a^ | 1.33±0.26^a^ | |
|  | H2H2 (8) | 0.45±0.42^c^ | 5.11±0.42^cd^ | 1.90±0.42^c^ | 3.35±0.42^bc^ | 4.72±0.42^bc^ | 0.16±0.42^b^ | 0.89±0.42 | 0.64±0.42^bc^ | -0.03±0.42^b^ | |
|  | H2H3 (19) | 2.07±0.26^ab^ | 6.03±0.26^ab^ | 4.10±0.26^b^ | 4.37±0.26^a^ | 5.48±0.26^ab^ | 1.36±0.26^a^ | 1.59±0.26 | 1.43±0.26^abc^ | 1.01±0.26^a^ | |
|  | H3H3 (2) | -95.0±0.26^d^ | 3.94±0.26^d^ | -93.8±0.26^d^ | 1.84±0.26^c^ | 3.68±0.26^c^ | -95.3±0.26^c^ | 0.29±0.26 | -0.96±0.26^c^ | -95.3±0.26^c^ | |
|  | *p* Value | < 0.0001** | 0.0004** | < 0.0001** | 0.0022** | 0.0019** | < 0.0001** | 0.3835 | 0.0024** | < 0.0001** | |
| Block2 | H1H1 (77) | 2.50±0.20^a^ | 6.14±0.20^a^ | 4.50±0.20^a^ | 4.29±0.20^a^ | 5.58±0.20^a^ | 1.72±0.20^a^ | 1.66±0.20^a^ | 1.69±0.20^a^ | 1.39±0.20^a^ | |
|  | H1H2 (14) | 1.02±0.32^c^ | 5.34±0.32^bc^ | 2.61±0.32^d^ | 3.68±0.32^bc^ | 4.90±0.32^bc^ | 0.65±0.32^bc^ | 1.27±0.32^ab^ | 1.08±0.32^bc^ | -0.57±0.32^d^ | |
|  | H1H3 (10) | 2.32±0.36^a^ | 6.38±0.36^a^ | 4.49±0.36^a^ | 4.31±0.36^ab^ | 6.08±0.36^a^ | 1.48±0.36^ab^ | 1.12±0.36^ab^ | 1.82±0.36^ab^ | 1.22±0.36^ab^ | |
|  | H2H2 (23) | 1.50±0.28^bc^ | 4.95±0.28^c^ | 3.57±0.28^bc^ | 2.98±0.28^d^ | 4.33±0.28^c^ | 0.65±0.28^c^ | 0.93±0.28^b^ | 0.54±0.28^c^ | 0.55±0.28^bc^ | |
|  | H2H3 (14) | 1.93±0.33^ab^ | 5.83±0.33^ab^ | 4.08±0.33^ab^ | 3.06±0.33^cd^ | 5.44±0.33^ab^ | 0.95±0.33^bc^ | 0.99±0.33^b^ | 1.19±0.33^abc^ | 0.58±0.33^bc^ | |
|  | H3H3 (14) | 1.30±0.33^bc^ | 5.28±0.33^bc^ | 3.08±0.33^cd^ | 3.11±0.33^cd^ | 4.88±0.33^bc^ | 0.77±0.33^bc^ | 0.90±0.33^b^ | 0.99±0.33^bc^ | 0.16±0.33^cd^ | |
|  | *p* Value | < 0.0001** | < 0.0001** | < 0.0001** | < 0.0001** | < 0.0001** | < 0.0001** | 0.0151* | 0.0002** | | < 0.0001** |
| 24 h after mixing | | | | | | | | | | | |
| Block1 | H1H1 (55) | 3.48±0.23^a^ | 7.15±0.23^a^ | 5.64±0.23^a^ | 5.11±0.23 | 6.70±0.23^a^ | 2.50±0.23^a^ | 2.90±0.23 | 2.48±0.23^a^ | | 2.32±0.23^a^ |
|  | H1H2 (41) | 3.21±0.24^a^ | 6.81±0.24^ab^ | 5.00±0.24^b^ | 4.77±0.24 | 6.42±0.24^ab^ | 2.66±0.24^a^ | 2.71±0.24 | 2.58±0.24^a^ | | 2.36±0.24^a^ |
|  | H1H3 (26) | 3.45±0.26^a^ | 7.09±0.26^a^ | 5.36±0.26^ab^ | 4.98±0.26 | 6.67±0.26^a^ | 2.79±0.26^a^ | 2.51±0.26 | 2.69±0.26^a^ | | 2.36±0.26^a^ |
|  | H2H2 (8) | 2.04±0.40^b^ | 6.21±0.40^b^ | 4.19±0.40^c^ | 4.78±0.40 | 5.52±0.40^b^ | 1.04±0.40^b^ | 2.59±0.40 | 1.48±0.40^b^ | | 2.02±0.40^a^ |
|  | H2H3 (19) | 3.25±0.26^a^ | 6.93±0.26^ab^ | 5.32±0.26^ab^ | 5.02±0.26 | 6.46±0.26^a^ | 2.41±0.26^a^ | 2.52±0.26 | 2.27±0.26^ab^ | | 2.17±0.26^a^ |
|  | H3H3 (2) | -94.9±0.26^c^ | 5.46±0.74^b^ | -94.2±0.26^d^ | 3.93±0.76 | 5.11±0.75^b^ | -95.3±0.26^c^ | 1.89±0.75 | 1.09±0.74^b^ | | -95.0±0.26^b^ |
|  | *p* Value | < 0.0001** | 0.0382* | < 0.0001** | 0.4647 | 0.0207* | < 0.0001** | 0.4481 | 0.0289* | | < 0.0001** |
| Block2 | H1H1 (77) | 3.53±0.20^a^ | 7.12±0.20^a^ | 5.53±0.20^a^ | 5.10±0.20^a^ | 6.65±0.20^a^ | 2.75±0.20^a^ | 2.77±0.20 | 2.62±0.20^a^ | | 2.51±0.20^a^ |
|  | H1H2 (14) | 2.92±0.32^b^ | 6.78±0.32^ab^ | 4.76±0.32^b^ | 4.98±0.32^ab^ | 6.34±0.32^a^ | 2.34±0.32^ab^ | 2.45±0.32 | 2.38±0.32^ab^ | | 1.73±0.32^b^ |
|  | H1H3 (10) | 3.02±0.36^ab^ | 6.98±0.36^ab^ | 5.14±0.36^ab^ | 5.07±0.36^ab^ | 6.78±0.36^a^ | 2.18±0.36^ab^ | 2.74±0.36 | 2.41±0.36^ab^ | | 2.07±0.36^ab^ |
|  | H2H2 (23) | 2.96±0.27^b^ | 6.34±0.27^b^ | 5.02±0.27^b^ | 4.60±0.27^ab^ | 5.62±0.27^b^ | 2.12±0.27^b^ | 2.40±0.27 | 1.85±0.27^b^ | | 2.10±0.27^ab^ |
|  | H2H3 (14) | 2.78±0.33^b^ | 6.92±0.33^ab^ | 4.95±0.33^b^ | 4.29±0.33^b^ | 6.71±0.33^a^ | 1.74±0.33^b^ | 2.44±0.33 | 1.98±0.33^b^ | | 1.87±0.33^b^ |
|  | H3H3 (14) | 2.34±0.33^b^ | 6.32±0.33^b^ | 3.97±0.33^c^ | 4.50±0.33^ab^ | 5.98±0.33^ab^ | 1.92±0.33^b^ | 2.27±0.33 | 2.01±0.33^b^ | | 1.37±0.33^b^ |
|  | *p* Value | 0.0010** | 0.0160* | < 0.0001** | 0.0479* | 0.0007** | 0.0026** | 0.4353 | 0.0201* | | 0.0018** |
| 48 h after mixing | | | | | | | | | | | |
| Block1 | H1H1 (55) | 3.61±0.23^a^ | 7.25±0.23^a^ | 5.73±0.23^a^ | 5.23±0.23 | 6.79±0.23^a^ | 2.70±0.23^a^ | 3.04±0.23 | 2.61±0.23^ab^ | | 2.47±0.23^a^ |
|  | H1H2 (41) | 3.31±0.24^ab^ | 6.88±0.24^ab^ | 5.13±0.23^b^ | 4.95±0.24 | 6.42±0.24^ab^ | 2.73±0.24^a^ | 2.80±0.24 | 2.65±0.24^ab^ | | 2.43±0.24^a^ |
|  | H1H3 (26) | 3.58±0.26^ab^ | 7.19±0.26^a^ | 5.49±0.26^ab^ | 5.22±0.26 | 6.71±0.26^a^ | 2.91±0.26^a^ | 2.74±0.26 | 2.74±0.26^a^ | | 2.53±0.26^a^ |
|  | H2H2 (8) | 2.80±0.41^b^ | 6.51±0.40^ab^ | 4.90±0.41^b^ | 4.96±0.41 | 5.65±0.41^b^ | 1.89±0.41^bc^ | 2.79±0.41 | 1.88±0.41^bc^ | | 2.33±0.41^a^ |
|  | H2H3 (19) | 3.38±0.26^ab^ | 7.01±0.26^ab^ | 5.39±0.26^ab^ | 5.10±0.26 | 6.56±0.26^a^ | 2.61±0.26^ab^ | 2.61±0.26 | 2.37±0.26^abc^ | | 2.33±0.26^a^ |
|  | H3H3 (2) | 0.88±0.74^c^ | 5.53±0.74^b^ | 1.88±0.74^c^ | 4.31±0.74 | 4.99±0.74^b^ | 0.71±0.74^c^ | 2.33±0.74 | 1.01±0.74^c^ | | -95.4±0.74^b^ |
|  | *p* Value | < 0.0029** | 0.0633* | < .0001** | 0.6094 | 0.0137* | 0.0210* | 0.6140 | 0.0850 | | < 0.0001** |
| Block2 | H1H1 (77) | 3.64±0.19^a^ | 7.22±0.19^a^ | 5.64±0.19^a^ | 5.25±0.19 | 6.73±0.19^a^ | 2.88±0.19^a^ | 2.89±0.19 | 2.72±0.19^a^ | | 2.62±0.19^a^ |
|  | H1H2 (14) | 3.00±0.31^b^ | 6.87±0.31^ab^ | 4.90±0.31^b^ | 5.03±0.31 | 6.45±0.31^ab^ | 2.36±0.31^ab^ | 2.56±0.31 | 2.43±0.31^ab^ | | 1.85±0.31^b^ |
|  | H1H3 (10) | 3.09±0.36^bc^ | 6.95±0.37^ab^ | 5.18±0.36^ab^ | 5.14±0.36 | 6.75±0.36^ab^ | 2.27±0.36^ab^ | 2.76±0.36 | 2.41±0.36^ab^ | | 2.15±0.36^ab^ |
|  | H2H2 (23) | 3.16±0.27^ab^ | 6.46±0.27^b^ | 5.17±0.27^ab^ | 4.76±0.27 | 5.66±0.27^c^ | 2.38±0.27^b^ | 2.64±0.27 | 1.96±0.27^b^ | | 2.33±0.27^ab^ |
|  | H2H3 (14) | 3.08±0.33^ab^ | 7.09±0.34^ab^ | 5.17±0.33^ab^ | 4.57±0.33 | 6.84±0.33^a^ | 2.16±0.33^b^ | 2.56±0.33 | 2.18±0.33^ab^ | | 2.21±0.33^ab^ |
|  | H3H3 (14) | 2.71±0.33^b^ | 6.45±0.33^b^ | 4.45±0.33^b^ | 4.69±0.33 | 6.03±0.33^bc^ | 2.22±0.33^b^ | 2.41±0.33 | 2.10±0.33^b^ | | 1.70±0.33^b^ |
|  | *p* Value | 0.0128* | 0.0211* | 0.0022** | 0.1112 | 0.0004** | 0.0330* | 0.5559 | 0.0296* | | 0.0169* |
| 72 h after mixing | | | | | | | | | | | |
| Block1 | H1H1 (55) | 3.66±0.23^a^ | 7.31±0.23^a^ | 5.76±0.23^a^ | 5.34±0.23 | 6.83±0.23^a^ | 2.78±0.23^a^ | 3.41±0.23^a^ | 2.72±0.23^a^ | | 2.55±0.23^a^ |
|  | H1H2 (41) | 3.35±0.24^ab^ | 6.93±0.24^ab^ | 5.16±0.24^b^ | 5.00±0.24 | 6.45±0.24^ab^ | 2.78±0.24^ab^ | 2.88±0.24^b^ | 2.68±0.24^a^ | | 2.47±0.24^a^ |
|  | H1H3 (26) | 3.61±0.26^ab^ | 7.22±0.26^a^ | 5.51±0.26^ab^ | 5.26±0.26 | 6.73±0.26^a^ | 2.95±0.26^a^ | 2.97±0.26^ab^ | 2.76±0.26^a^ | | 2.54±0.26^a^ |
|  | H2H2 (8) | 2.87±0.40^b^ | 6.61±0.40^ab^ | 4.92±0.40^b^ | 5.15±0.40 | 5.69±0.40^b^ | 2.00±0.40^bc^ | 2.85±0.40^b^ | 1.94±0.40^ab^ | | 2.45±0.40^a^ |
|  | H2H3 (19) | 3.44±0.26^ab^ | 7.06±0.26^a^ | 5.42±0.26^ab^ | 5.14±0.26 | 6.60±0.26^a^ | 2.71±0.26^ab^ | 3.08±0.26^ab^ | 2.40±0.26^ab^ | | 2.39±0.26^a^ |
|  | H3H3 (2) | 0.88±0.74^c^ | 5.55±0.74^b^ | 1.89±0.74^c^ | 4.35±0.74 | 5.02±0.74^b^ | 0.71±0.74^c^ | 2.02±0.74^b^ | 0.97±0.74^b^ | | -95.4±0.74^b^ |
|  | *p* Value | 0.0027** | 0.0646 | < 0.0001** | 0.5418 | 0.0133* | 0.0228* | 0.0779 | 0.0618 | | < 0.0001** |
| Block2 | H1H1 (77) | 3.68±0.19^a^ | 7.28±0.19^a^ | 5.66±0.19^a^ | 5.32±0.19 | 6.78±0.19^a^ | 2.94±0.19^a^ | 3.26±0.19 | 2.79±0.19^a^ | | 2.66±0.19^a^ |
|  | H1H2 (14) | 3.01±0.32^b^ | 6.90±0.32^ab^ | 4.92±0.32^b^ | 5.03±0.32 | 6.50±0.32^ab^ | 2.35±0.32^ab^ | 2.63±0.32 | 2.41±0.32^ab^ | | 1.89±0.32^bc^ |
|  | H1H3 (10) | 3.14±0.36^ab^ | 6.99±0.36^ab^ | 5.20±0.36^ab^ | 5.15±0.36 | 6.81±0.36^ab^ | 2.35±0.36^ab^ | 3.07±0.36 | 2.37±0.36^ab^ | | 2.20±0.36^abc^ |
|  | H2H2 (23) | 3.27±0.27^ab^ | 6.61±0.27^b^ | 5.22±0.27^ab^ | 4.92±0.27 | 5.80±0.27^b^ | 2.58±0.27^ab^ | 3.19±0.27 | 2.02±0.27^b^ | | 2.49±0.27^ab^ |
|  | H2H3 (14) | 3.18±0.33^ab^ | 7.17±0.33^ab^ | 5.22±0.33^ab^ | 4.75±0.33 | 6.89±0.33^a^ | 2.36±0.33^ab^ | 2.88±0.33 | 2.17±0.34^b^ | | 2.39±0.33^abc^ |
|  | H3H3 (14) | 2.79±0.33^b^ | 6.56±0.33^b^ | 4.51±0.33^b^ | 4.71±0.33 | 6.12±0.33^ab^ | 2.31±0.33^b^ | 2.85±0.33 | 2.17±0.33^b^ | | 1.78±0.33^c^ |
|  | *p* Value | 0.0205* | 0.0465* | 0.0033** | 0.1970 | 0.0013** | 0.0769 | 0.2886 | 0.0248* | | 0.0236* |

Note: *P*-value shows the significance for genetic effects among the haplotype blocks; ^abcd^ within the same column with different superscripts means significant differences, and the same superscript indicates no significant differences. Block 1: H1 = GGAA, H2 = TAGC, H3 = GAAA. Block 2: H1 = AAC, H2 = GAA, H3 = GTA.
