# Supplementary material for: A Single-Nucleotide Polymorphism in the Promoter of Porcine ARHGAP24 Gene Regulates Aggressive Behavior of Weaned Pigs After Mixing by Affecting the Binding of Transcription Factor p53
Source: Front Cell Dev Biol. 2022 Apr 1;10:839583. doi: 10.3389/fcell.2022.839583 (PMC9010951; doi:10.3389/fcell.2022.839583)
Supplement: Supplementary file 7 [file Table2.DOC]

# Supplementary Tables

**Table S2.** Information on of plasmid primer construction and RT-qPCR for the porcine *ARHGAP24* gene.

| Primer | Primer sequence (5'-3') | Product Size (bp) | Usage |
| --- | --- | --- | --- |
| ARHGAP24-P1 | F: 5’-CGACGCGTCGTCAGTTGTTCCAGCCTCTTGTA-3’  R: 5’-CCGCTCGAGCGGCCCAGGACAAGCCAAAAGAAA-3’ | 386 | Promoter vector Construction |
| ARHGAP24-P2 | F: 5’-CGACGCGTCGTGCGTGTCTATGCTATACCTGA-3’  R: 5’-CCGCTCGAGCGGCCCAGGACAAGCCAAAAGAAA-3’ | 642 | Promoter vector Construction |
| ARHGAP24-P3 | F: 5’-CGACGCGTCGTCTTCGGTTCACACTGGGTC-3’  R: 5’-CCGCTCGAGCGGCCCAGGACAAGCCAAAAGAAA-3’ | 1023 | Promoter vector Construction |
| ARHGAP24-P4 | F: 5’-CGACGCGTCGTTGATACATTAAAGGGTTGA-3’  R: 5’-CCGCTCGAGCGGCCCAGGACAAGCCAAAAGAAA-3’ | 1466 | Promoter vector Construction |
| ARHGAP24-P5 | F: 5’-CGACGCGTCGTCACAGACAGCAGGGGTGTT-3’  R: 5’-CCGCTCGAGCGGCCCAGGACAAGCCAAAAGAAA-3’ | 1925 | Promoter vector Construction |
| ARHGAP24-P6 | F: 5’-CGACGCGTCGAGGACAGACACCAACTCCCT-3’  R: 5’-CCGCTCGAGCGGCCCAGGACAAGCCAAAAGAAA-3’ | 2329 | Promoter vector Construction |
| ARHGAP24-Haplotype | F: 5’-CGACGCGTCGATCACCAGGAAGAAAGAC-3’  R: 5’-CCGCTCGAGCGGAGAGAAATTCAAAGCATG-3’ | 439 | Construction of eukaryotic expression vectors with different haplotypes |
| PBEs | F: 5’-AGAGCCCTAATGAGCACA-3’  R: 5’-CTTCCCACCAGCAGAAAA-3’ | 168 | ChIP |
| Site X | F: 5’-GTCCTCTTCGGTTCACAC-3’  R: 5’-CAATCAGAATTAGCTGGG-3’ | 156 | ChIP |
| ARHGAP24-PBE-WT | F: 5’-CGACGCGTCGTGTGAAACCATCTAAAGCA-3’  R: 5’-CCGCTCGAGCGGGACCCAGTGTGAACCGAA-3’ | 174 | Vector with promoter of wild p53 binding motif |
| ARHGAP24-PBE-MUT | F: 5’-GTACTGTCCTGTAACTTTTTGAAAGAGACCTTTTCTGCTGG-3’  R: 5’-AAGTTACAGGACAGTACCGGTTACCTGGAACA-3’ |  | Vector with promoter of mutant p53 binding motif |
| CDS-p53 | F: 5’-CCGGAATTCCGGACAGTGACACGCTCTCCTGA-3’  R: 5’-CCGCTCGAGCGGCACCAAGCAGAGGTCTAAGCA-3’ | 1290 | Construction of eukaryotic expression vector of p53 |
| P53-siRNA | F: 5’-GCCACCUGAAGUCUAAGAATT-3’  R: 5’-UUCUUAGACUUCAGGUGGCTT-3’ |  | siRNA primer for p53 |
| ARHGAP24-siRNA | F: 5’-GGAGGAUACUGUCCGUUAUTT-3’  R: 5’-AUAACGGACAGUAUCCUCCTT-3’ |  | siRNA primer for ARHGAP24 |
| ARHGAP24 | F: 5’-AGCGGAGGCGTCTGATGTTAG-3’  R: 5’-CAAACCAACGTGTATGCCAGG-3’ | 187 | RT-qPCR |
| P53 | F: 5’-CCCCTCCCCAGCATCTCA-3’  R: 5’-CACAAACACGCACCTCAAAG-3’ | 261 | RT-qPCR |
| RHOA | F: 5’-ATGGAAAGCAGGTAGAGTTG-3’  R: 5’-ACCAGGATGATGGGCACA-3’ | 199 | RT-qPCR |
| ROCK1 | F: 5’-AACAATCCAATCCGTCTA-3’  R: 5’-ACTGGTTCCATCTCTACG-3’ | 166 | RT-qPCR |
| RAC1 | F: 5’-GTGTGTGGTGGTGGGAGA-3’  R: 5’-GTCAAAGACCGTGGGGAT-3’ | 100 | RT-qPCR |
| GAPDH | F: 5’-CCACGGTCCATGCCATCACT-3’  R: 5’-GCCTGCTTCACCACCTTCTTG-3’ | 268 | RT-qPCR |

Note: underline type is protective bases; italic type is enzyme digestion site, MluI (ACGCGT), XhoI (CTCGAG), EcoRI (GAATTC).
